# Sulfidation and Reoxidation of U(VI)-Incorporated Goethite: Implications for U Retention during Sub-Surface Redox Cycling

**DOI:** 10.1021/acs.est.2c05314

**Published:** 2022-11-30

**Authors:** Olwen Stagg, Katherine Morris, Luke Thomas Townsend, Kristina O. Kvashnina, Michael L. Baker, Ryan L. Dempsey, Liam Abrahamsen-Mills, Samuel Shaw

**Affiliations:** † Research Centre for Radwaste Disposal and Williamson Research Centre for Molecular Environmental Science, Department of Earth and Environmental Sciences, The University of Manchester, ManchesterM13 9PL, U.K.; ‡ The Rossendorf Beamline at ESRF—The European Synchrotron, CS40220, Grenoble Cedex 938043France; § Institute of Resource Ecology, Helmholtz Zentrum Dresden Rossendorf (HZDR), Dresden01314, Germany; ∥ Department of Chemistry, The University of Manchester, ManchesterM13 9PL, U.K.; ⊥ The University of Manchester at Harwell, The University of Manchester, Diamond Light Source, Harwell Campus, DidcotOX11 0DE, U.K.; # National Nuclear Laboratory, WarringtonWA3 6AE, Cheshire, U.K.

**Keywords:** sulfidation, uranium, iron (oxyhydr)oxides, XAS, persulfide

## Abstract

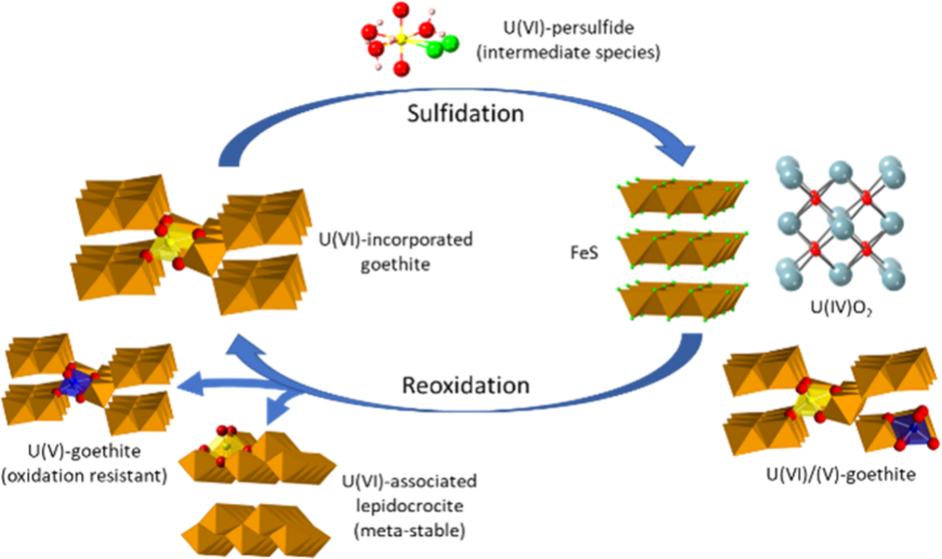

Over 60 years of nuclear activity have resulted in a global legacy of contaminated land and radioactive waste. Uranium (U) is a significant component of this legacy and is present in radioactive wastes and at many contaminated sites. U-incorporated iron (oxyhydr)oxides may provide a long-term barrier to U migration in the environment. However, reductive dissolution of iron (oxyhydr)oxides can occur on reaction with aqueous sulfide (sulfidation), a common environmental species, due to the microbial reduction of sulfate. In this work, U(VI)–goethite was initially reacted with aqueous sulfide, followed by a reoxidation reaction, to further understand the long-term fate of U species under fluctuating environmental conditions. Over the first day of sulfidation, a transient release of aqueous U was observed, likely due to intermediate uranyl(VI)–persulfide species. Despite this, overall U was retained in the solid phase, with the formation of nanocrystalline U(IV)O_2_ in the sulfidized system along with a persistent U(V) component. On reoxidation, U was associated with an iron (oxyhydr)oxide phase either as an adsorbed uranyl (approximately 65%) or an incorporated U (35%) species. These findings support the overarching concept of iron (oxyhydr)oxides acting as a barrier to U migration in the environment, even under fluctuating redox conditions.

## Abstract 2

Reaction of U(VI)−goethite with aqueous sulfide results in transient remobilization of trace levels of aqueous uranium with long-term solid phase retention of U(IV)O_2_.

## Introduction

Globally, U is considered a key environmental contaminant, prevalent in the sub-surface at numerous nuclear legacy and mining sites (e.g., Hanford/Rifle/Oak Ridge, USA).^1−4^ U is also significant in higher activity radioactive wastes that are destined for disposal in a deep underground geological disposal facility (GDF).^5^ To aid long-term containment, any GDF design will contain multiple barriers to limit radionuclide migration from the facility over geological timescales.^1,4,5^ In addition to naturally occurring minerals from the surrounding host rock of the GDF, the corrosion of engineering iron and steel structures will lead to iron (oxyhydr)oxide phases (e.g., magnetite, goethite, and green rust) being ubiquitous in and around the facility.^6−8^ Previous studies have shown that iron (oxyhydr)oxides can readily incorporate U species into their crystal structure and may therefore act as a further barrier to U migration in the environment over timescales relevant to a GDF.^9−23^ However, the sub-surface biogeochemistry of both contaminated land and GDF environments will evolve over time, and this may include redox cycling induced by the onset of sulfate-reducing conditions and/or by oxygen ingress.^1,24−26^ Consequently, U-associated iron (oxyhydr)oxide phases may react with aqueous sulfide via a sulfidation reaction.^27,28^ Potential reoxidation of this sulfidized system may then occur over the longer term, with cycling likely between reduced and oxidized states.^29,30^ Given the potential for these fluctuating biogeochemical cycles in the sub-surface (e.g., effects of redox cycling, organic matter, carbonates, etc),^31,32^ the long-term fate of incorporated radionuclides (including U) is unclear.

Under environmental sub-surface conditions, the migration of U species is often dominated by changes in the redox potential, with oxidation state a major control on U mobility.^2^ Under circumneutral conditions in the sub-surface, U generally exists as either U(VI) or U(IV) under oxic and anoxic conditions, respectively.^1,2,33^ U(VI) typically forms the relatively mobile uranyl ion (UO_2(aq)_^2+^), whereas U(IV) may form poorly soluble phases of either non-crystalline U(IV) or nanoparticulate uraninite (UO_2_).^1,2,33^ In addition, although U(V) can undergo disproportionation to U(IV) and U(VI),^34^ recent studies have indicated that U(V) may be formed and stabilized during a number of biogeochemical processes in the environment.^35,36^ In particular, U(V) can be stabilized on incorporation into iron (oxyhydr)oxide phases,^13,15,16,18−21^ and U(VI) incorporated within iron (oxyhydr)oxides may undergo reduction to U(V).^13,23^

The formation of U-incorporated iron (oxyhydr)oxide phases is thought to occur via substitution of U(VI/V) for an Fe(III) within the mineral structure, potentially immobilizing U in the long-term.^12,13,15,16,18−21,23,37^ Iron (oxyhydr)oxide mineral phases are ubiquitous in engineered and natural environments, commonly forming from the breakdown of Fe-containing silicate minerals (e.g., olivine) by the oxidation of dissolved ferrous iron and during metal corrosion in engineered systems (e.g., contaminated land and GDF scenarios).^38,39^ Iron (oxyhydr)oxides in engineered and natural environments may be subjected to fluctuating redox conditions, such as oxygen ingress or the onset of microbially mediated iron reducing conditions.^2,40−42^ Additionally, in many sub-surface scenarios (e.g., organic-rich sediments), microbial sulfate reduction may occur, in turn producing aqueous sulfide species.^1,24,43,44^ The resulting sulfide may then react with iron (oxyhydr)oxide phases via a process known as sulfidation. Sulfidation of iron (oxyhydr)oxides is a complex multi-step process,^28^ which typically results in reductive dissolution at the mineral surface, forming elemental sulfur (S_8_^0^) and releasing Fe(II) into solution.^27,45^ The resultant aqueous Fe(II) may then react with aqueous HS^–^ to form secondary iron sulfide phases, such as mackinawite (FeS).^27,45^ Aqueous HS^–^ may also react with S_8_^0^ to form surface-associated polysulfide (S_*n*_^2–^) species.^46^ Consequently, these complex processes may influence the behavior and fate of radionuclides, including U.^26,47−50^

A field study at Rifle (USA) observed a release of aqueous U following the onset of microbial sulfate reduction.^24^ Transient aqueous U release was also observed during abiotic sulfidation reactions, including the reaction of an abiotic sulfide solution with U(V)-incorporated magnetite,^51^ U(VI)-adsorbed hematite, and U(VI)-adsorbed lepidocrocite.^52,53^ These abiotic studies suggested that U release may be a result of poor U(VI) affinity for a sulfidized surface.^51−53^ Recently, further insight was provided by the sulfidation of U(VI)-adsorbed ferrihydrite, where aqueous U(VI) speciation was attributed to the formation of an intermediate uranyl(VI)–persulfide species, which had only a weak adsorption affinity for FeS.^54^ However, in all abiotic sulfidation systems, overall reduction to U(IV) was observed, with U being retained mainly as either nanoparticulate uraninite (U(IV)O_2_)^51,53,54^ or non-crystalline U(IV).^52^

On exposure of U(IV)O_2_ to oxygen, oxidation to U(VI) typically occurs.^29,42^ However, nanocrystalline mackinawite (FeS) has previously been shown to protect U(IV)O_2_ from reoxidation;^55^ synthetic nanocrystalline FeS acted as an oxygen scavenger, transforming to nanogoethite and lepidocrocite, with no U(IV)O_2_ dissolution observed prior to complete FeS depletion.^55^ U(IV)O_2_ oxidative dissolution, under carbonate rich conditions (5% CO_2_/2% O_2_ gas mixture, 4 mM NaHCO_3_), then led to the remobilization of aqueous U(VI)–carbonato complexes and subsequent U(VI) adsorption (25%) onto goethite/lepidocrocite. Consequently, following sulfidation, the long-term fate of U(IV)O_2_ will be dependent on the oxygen scavenging capability of the formed FeS phase and the ambient physicochemical conditions, with carbonate concentration being a significant control.^55^ Simultaneous reoxidation of FeS and U(IV)O_2_ may result in partial U(V,VI) incorporation into the forming iron (oxyhydr)oxide phase. Alternatively, FeS may scavenge oxygen, as already observed with nanocrystalline mackinawite,^55^ thereby delaying U(IV)O_2_ reoxidation and potentially preventing U(V,VI) incorporation into newly formed Fe(III)-bearing (oxyhydr)oxides. This may lead to delayed U(IV) oxidation, presumptively to U(VI), which would then likely be retained as a more labile adsorbed phase with potentially higher mobility in the environment.

Given the observed transient release of U during the sulfidation of U-associated magnetite,^51^ ferrihydrite,^54^ hematite, and lepidocrocite^52,53^ for iron (oxyhydr)oxides to be considered as long-term sequesters of U in sub-surface environmental systems, further understanding of U behavior during sulfidation and associated redox cycling (e.g., reoxidation) is needed. In particular, the mechanism for the transient U release during iron (oxyhydr)oxide sulfidation is still unclear, as is the long-term fate of the reduced U(IV)O_2_ phase formed during sulfidation. Here, highly controlled sulfidation and air reoxidation experiments were performed on U(VI)-incorporated goethite using a chemostat system.^51,54^ In contaminated land and waste disposal scenarios, dissolved sulfide may originate from microbial reduction of sulfate or from groundwater sources.^24,26^ The rate of sulfide ingress in sub-surface systems is consequently controlled by groundwater flow or in situ biotic formation. Therefore, to make this system more environmentally relevant, sulfide was slowly added to the experiment over 4 h at a constant rate,^51,54^ with an aqueous sulfide concentration similar to that measured in anoxic sediments.^56^ Throughout, reactions were monitored at selected timepoints using geochemical analyses (e.g., ICP–MS and colorimetric assay^57^), X-ray absorption spectroscopy (XAS), and transmission electron microscopy (TEM).

## Materials and Methods

### Mineral Preparation

U(VI)-incorporated goethite (approximately 0.2 wt % U) was formed via a hydrothermal synthesis method, as previously described.^23^ The resultant slurry was then washed several times with DIW and washed with 4 mM HCl to remove adsorbed U(VI).^58,59^ After several more washes with DIW, the solid was left to dry overnight (40 °C), and the mineral phase was confirmed by powder X-ray diffraction (XRD).

### Sulfidation Experiment

Experiments were performed under anoxic conditions in an Applikon Bioreactor (nitrogen atmosphere), which monitored and/or controlled the pH, Eh, temperature, and reagent additions as required.^54^ Samples were periodically collected under anoxic conditions, with all sample manipulations conducted within an anaerobic Coy cabinet under a mixed nitrogen/hydrogen (95%:5%) atmosphere. A U(VI)–goethite slurry was prepared (400 mL, 1 g/L) and transferred to the Applikon Bioreactor under a flow of N_2_ and then left to equilibrate overnight. A sodium sulfide solution (0.4 M) was prepared in the Coy cabinet from sodium sulfide nonahydrate (Na_2_S·9H_2_O), with the concentration confirmed through the methylene blue assay using the radiello RAD171 standard.^57^ The resulting sodium sulfide solution was then added to the vessel at a constant rate (0.1 mL/min) over 4 h to reach a final total S(−II)/Fe(III) molar ratio of 2:1. The experiment was kept anoxic under a constant flow of N_2_, and the pH was maintained at pH 7 via the automated addition of 1 M HCl. The reaction was controlled in the chemostat vessel for 72 h and then transferred to a Schott bottle in the Coy cabinet for long-term anaerobic storage. During sulfidation, the experiment was sampled periodically, and the slurry was filtered to <1.5 nm using 3 kDa Nanosep Centrifugation Ultrafilters (PES). The filtrate was then preserved for analysis by either acidification for cation analysis (U and Fe) or by reaction with a zinc acetate solution (82 mM) for sulfide analysis. Aqueous Fe and U were monitored by inductively coupled plasma mass spectrometry (ICP–MS, Agilent 8900), with aqueous sulfide analyzed using the methylene blue assay and the radiello RAD171 standard.^57^

For solid-phase analysis, samples were studied by TEM, conventional XAS, and XAS in a high energy resolution detection (HERFD) mode. For TEM, sample slurry was dropped onto TEM grids (holey C film on Au 300 mesh) and dried inside the anaerobic cabinet prior to analysis on either FEI Tecnai TF20 or FEI Titan3 Themis 300 (LEMAS). For XAS analysis, solid samples were obtained by filtration (nylon membrane filter, 0.22 μm) and then stored and transported at −80 °C under anoxic conditions to the Diamond Light Source (UK) for analysis on either the I20-scanning or B18 beamline; for HERFD-XANES, select solid filtrate samples were transported frozen and under anoxic conditions to the European Synchrotron Radiation Facility (ESRF) in Grenoble (further details in Section S3).

### Reoxidation Experiment

As with the sulfidation study, the reoxidation experiment was also performed in the Applikon Bioreactor, which again monitored and/or controlled the dissolved oxygen (DO), pH, Eh, temperature, and reagent additions. After sampling, sample manipulations were conducted within an anaerobic Coy cabinet. Briefly, after 5 months of aging under anoxic conditions, the sulfidized U(VI)–goethite slurry was diluted in de-oxygenated water (0.5 g/L, 200 mL), transferred to the Applikon Bioreactor, and left to equilibrate overnight under a flow of nitrogen. The pH was maintained at pH 7 via addition of 0.05 M HCl or 0.05 M NaOH, and the reaction was initiated by the introduction of laboratory air, with the chemostat set to maintain a DO level of 5% within the solution. Samples were collected periodically over 4 days, with aqueous (U and Fe) and solid phase (XRD, TEM, and XAS) samples collected as above for analysis.

## Results and Discussion

The U(VI)-incorporated goethite was initially characterized by XRD (Figure S7) to confirm that goethite was the only crystalline phase present. Previous analysis has confirmed that U(VI) was incorporated within the goethite structure by substitution into an Fe(III) site (∼0.2 wt % U), forming a distorted octahedral coordination.^23^

### Sulfidation of U(VI)–Goethite

**Figure 1:**
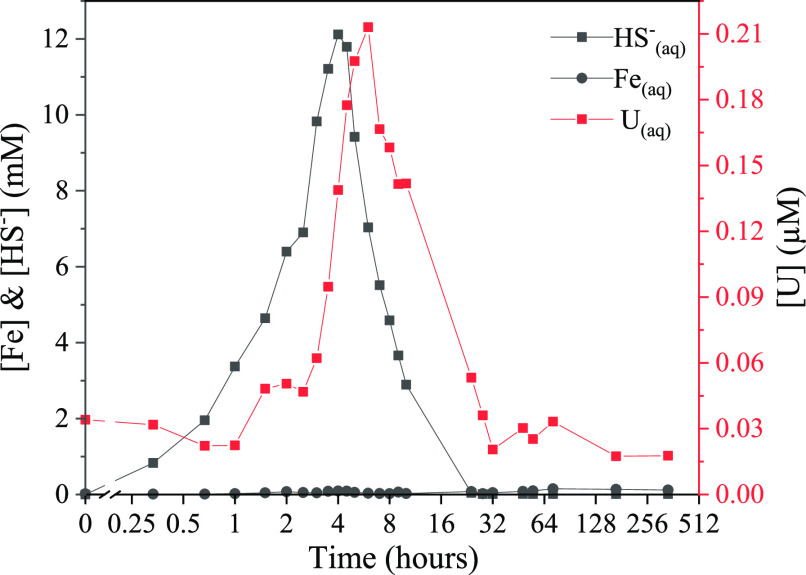
Aqueous Fe, HS^–^, and U during the sulfidation of U(VI)-incorporated goethite. The *x*-axis is shown as log_2_ after 0 h.

Sulfidation of the U(VI)–goethite slurry (1 g/L, 11.3 mM Fe) was initiated by a controlled 4 h addition of aqueous sulfide (22.5 mM) to reach a final total S(−II)/Fe(III) molar ratio of 2:1. Over the 4 h of addition, the concentration of aqueous sulfide increased steadily to a peak of 12.1 mM at 4 h (Figure 1), followed by a gradual decrease in aqueous concentration, with no detectable aqueous sulfide by 24 h. During this time, aqueous Fe (presumably as Fe(II)) followed the same increase in solution concentration at low but detectable levels, with a peak of 90.1 μM at 4 h (Figure S1). However, after aqueous sulfide had been removed (24 h), aqueous Fe steadily increased (150 μM by 72 h). These trends are characteristic of the reported sulfidation mechanism, with the steady decrease in aqueous sulfide from 4 h likely due to sulfide oxidation on reaction with, and concomitant reductive dissolution of, the U(VI)–goethite.^27,45,60^ However, given the excess aqueous sulfide in this system (HS^–^/Fe(III) molar ratio of 2:1), it is likely that immediate precipitation of FeS occurred following the release of Fe(II) into solution. This would explain the low levels of aqueous Fe initially detected, followed by an increase in aqueous Fe after all aqueous sulfide was removed from the system. As with previous studies of U associated with iron (oxyhydr)oxides,^51,52,54^ the reductive dissolution of U(VI)–goethite also displayed a transient release of U during sulfidation (Figure 1). Specifically, the aqueous U release followed the same trend as aqueous sulfide, albeit with a time delay of 1–2 h. Aqueous U increased steadily after 1 h, with a peak of 2.5% U_total_ (0.21 μM) at 6 h, followed by a gradual depletion over a further 26 h. As aqueous samples were collected using 3 kDa ultrafilters (approximately equivalent to 1.5 nm pore size), released U is assumed to be an aqueous U(VI) speciation (as opposed to colloidal U).^61,62^ Interestingly, past work ascribed the transient U release during U(VI)–ferrihydrite sulfidation to the formation of a uranyl(VI)–persulfide species.^54^

The mineral transformations that occurred during the sulfidation of U(VI)–goethite were monitored by TEM, with images collected at selected timepoints. After 1 day of sulfidation, sheet-like particles were observed that matched well with nanocrystalline mackinawite (FeS) morphology (Figure S8).^63^ In addition, XRD patterns collected at 4.5 h and 3 days (Figure S7) revealed a significant depletion in goethite over time, along with an ingress of poorly ordered FeS. Elemental sulfur (S_8_^0^), an important intermediate during sulfide oxidation in sub-surface systems,^64^ was also identified after 3 days (Figure S7). This confirms that rapid reductive dissolution of U(VI)–goethite, followed by secondary FeS formation, had occurred by 1 day. Furthermore, a selected area electron diffraction pattern collected after 7 months of aging (Figure S9) was also consistent with nanocrystalline FeS.^63^ However, the presence of persistent rod-shaped iron (oxyhydr)oxide phases can also be seen in the samples at 7 months (Figure S10), which are likely refractory U–goethite crystals. This is consistent with previous similar work that observed residual goethite particles after 6 months despite an initial excess of sulfide.^65^ Overall, TEM images confirm that although rapid sulfidation and reductive dissolution of U(VI)-incorporated goethite occurred, a residual U–goethite component was still present after 7 months.

**Figure 2:**
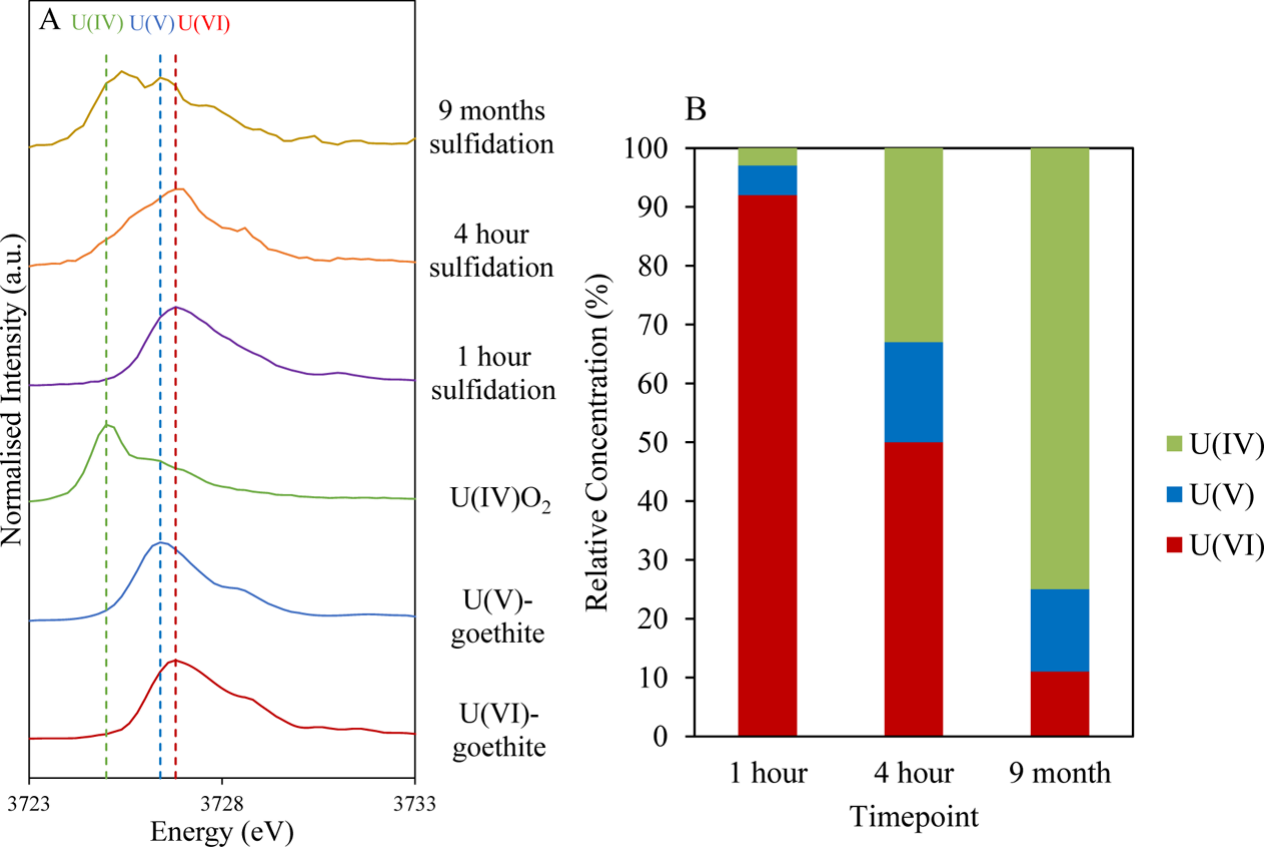
(A) U M_IV_-edge HERFD-XANES, showing timepoints for U(VI)–goethite sulfidation samples. Dashed lines indicate peaks for standards U(IV)O_2_, U(V)–goethite, and U(VI)–goethite. (B) Results from ITFA^66^ of U M_IV_-edge HERFD-XANES data, showing the relative concentrations of U(IV), U(V), and U(VI) in U(VI)–goethite sulfidation samples (further details in the Supporting Information, Table S1).

To monitor U speciation during U(VI)–goethite sulfidation, U M_IV_-edge HERFD-XANES and U L_III_-edge XAS data were collected at select timepoints. First, the change in U oxidation state was measured by U M_IV_-edge HERFD-XANES and further quantified using ITFA (full details in Table S1 and Figure S17).^66^ As expected, there was a continuous decrease in U(VI) and increase in U(IV) over time, from predominantly U(VI) after 1 h to predominantly U(IV) by 9 months (Figure 2). Interestingly, evidence for a U(V) component was also identified at 1 h; U(V) was a significant component by 4 h and was retained for up to 9 months (Figure 2; Table S1, Supporting Information). This seems to correlate with results from a recent study investigating the reduction of U(VI) by magnetite.^67^ On reaction with magnetite, U(VI) was initially reduced to a mixed U(IV)/U(V) oxide phase, with the formation of U(IV)O_2_ nanoparticles dominant by 4 weeks.^67^ In addition, the reduction of surface-associated U(VI) by FeS has previously been suggested to result in mixed valence U(IV,V) oxide phases, such as U_3_O_8_ or U_4_O_9_, as well as U(IV)O_2_.^49,50,68^ Therefore, we suggest that although U(IV)O_2_ formation may be dominant in the long-term (due to reducing agents such as Fe(II) or HS^–^), during the early stages of U(VI)–goethite sulfidation, surface-associated U(VI) on FeS may be reduced to mixed valence U(IV,V) bearing oxide particles (e.g., U_4_O_9_ or U_3_O_7_).

**Figure 3:**
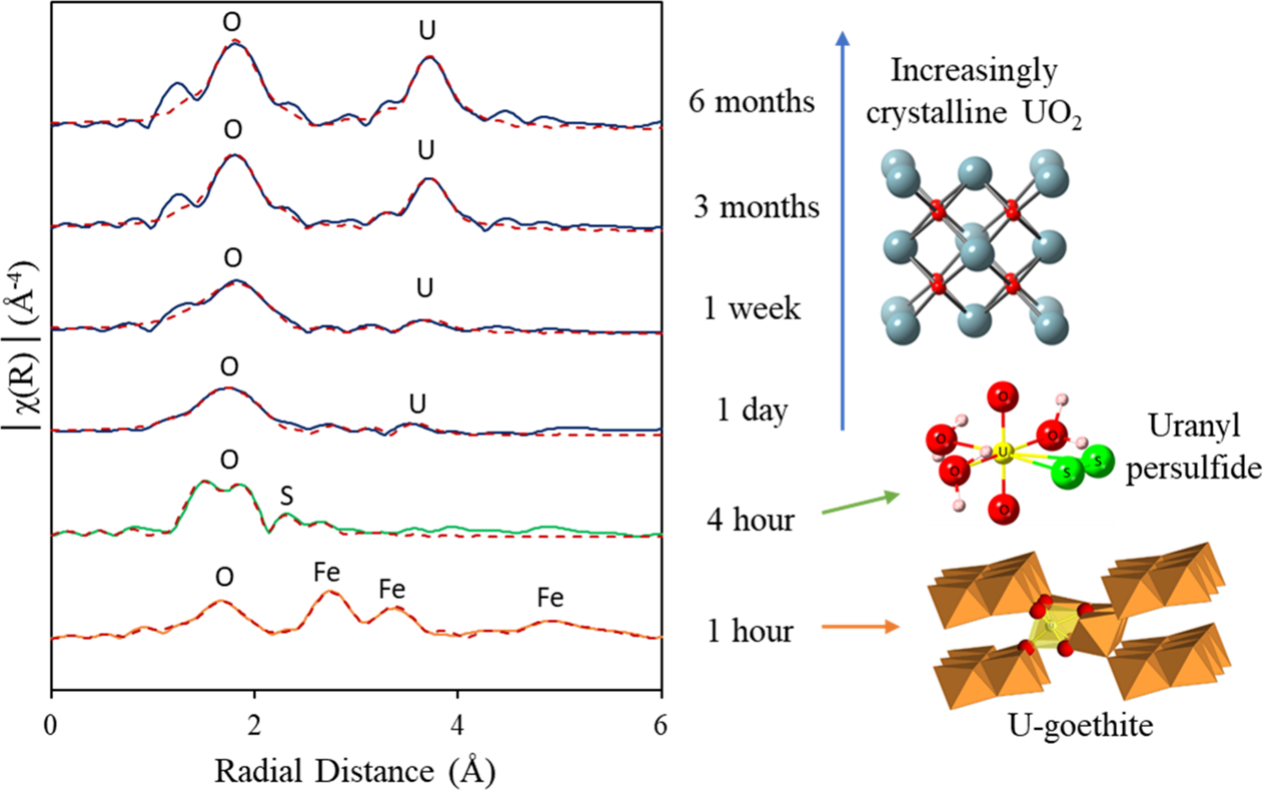
U L_III_-edge XAS spectra for U(VI)–goethite sulfidation, displaying the Fourier transform of *k*^3^-weighted EXAFS. Solid lines are data, and dashed lines are the modeled best fits.

U speciation was further probed by U L_III_-edge EXAFS to determine changes in the local coordination environment (Figure 3). After 1 h of sulfidation, 4 U–O and 7 U–Fe shells were fitted and tested using F-tests (Table S2, Supporting Information), with the fit matching closely to previous EXAFS studies on U(VI) incorporated into goethite (Table 1).^23^ Given that there was no aqueous U detected at 1 h (Figure 1), this suggests that U was still largely incorporated within the goethite structure. However, there was an elongation in U–O_1_ (1.82(1)–1.88(1) Å),^23^ possibly reflecting the formation of U(V), as indicated by the ITFA analysis of the U M_IV_-edge HERFD-XANES data (Figure 2). As U appears to be incorporated in goethite at this timepoint from the U–Fe shells still present in the EXAFS fit, this suggests that an electron transfer mechanism (from either adsorbed Fe(II) or HS^–^) may have partially reduced incorporated U(VI) to U(V) in goethite, as previously observed during the reaction of Fe(II) with U(VI)–goethite.^23^ By 4 h, the best fit had 3 U–O distances (1.85, 2.11, and 2.29 Å) and a S backscatterer (2.67 Å), with U–Fe shells (0.5 Fe backscatterers) at 3.21 and 3.44 Å. The diminished occupancy of the U–Fe shells indicates a decrease in a long range order surrounding the U, likely from significant reductive dissolution of U(VI)–goethite by 4 h (Figure 3). The presence of the small, but essential, number of S backscatterers in the fit (0.5 S at 2.67 Å; *F*-test = 100%, Supporting Information Table S2) indicated the presence of a uranyl persulfide species.^54^ Formation of the proposed uranyl persulfide species is likely favored by the uranyl adsorbing to persulfide anions (S_2_^2–^), which form at the goethite surface during sulfidation.^46^ In addition, density functional theory (DFT) calculations in past work suggested a weak adsorption affinity for uranyl(VI)–persulfide and the mackinawite surface.^54^ Therefore, the formation of a uranyl persulfide complex distributed between the solid and solution may explain the transient release of aqueous U during the first several hours of reaction. Interestingly, this transient uranyl persulfide species has only been observed during the sulfidation of U(VI) systems (here as U(VI)–goethite and during U(VI)–ferrihydrite sulfidation^54^) but was not observed during U(V)–magnetite sulfidation.^51^ In addition, the remobilization of U to solution was relatively short-lived in the circumneutral U(VI) systems (<32 h, Figure 1),^54^ yet for the U(V)–magnetite system, U remained in solution for over a week (complete removal by 9 days).^51^ A possible explanation for these observations could be related to the overall charge of the uranyl persulfide species. For U(VI), a uranyl ion (UO_2_^2+^) is bound to a disulfide species (S_2_^2–^) and coordinated by water molecules, resulting in a neutral complex that is relatively weakly bound to the FeS surface.^54^ This is thought to result in a short-lived transient aqueous U species, which is partially adsorbed onto the FeS surface, thereby enabling the uranyl(VI) persulfide species to be observed by EXAFS of the solid phase. However, a U(V) uranyl ion (UO_2_^+^) binding to a disulfide (S_2_^2–^) species would result in a negatively charged complex, as previously modeled using DFT calculations.^54^ As the point of zero charge for magnetite is 6.55,^69^ with nanomagnetite being the only identified phase (by TEM) after 8 h of reaction,^51^ a negatively charged U(V)–persulfide species would be repelled from the surface, not be associated with the solid EXAFS sample, and may persist in the solution phase for an extended period of time, as observed during U(V)–magnetite sulfidation.^51^ Overall, this suggests that although U(VI) and U(V) may both form an aqueous uranyl persulfide complex, the neutral U(VI) species is expected to be partially adsorbed to the solid phase at a circumneutral pH, and there is consequently only a transient release of U(VI) before reduction to U(IV) occurs.

**Table 1:** Details of the EXAFS Fits for the U(VI)–Goethite Standard and 1–4 h Sulfidation Samples (Full Details in the Supporting Information, Table S2)

U(VI)–goethite^a^			1 h			4 h		
path	*R* (Å)	CN	path	*R* (Å)	CN	path	*R* (Å)	CN
O_1_	1.82(1)	0.8	O_1_	1.88(1)	1	O_1_	1.85(1)	1
O_2_	2.03(2)	0.8	O_2_	2.06(3)	1	O_2_	2.11(2)	1.8
O_3_	2.23 (1)	2.2	O_3_	2.23(2)	2.5	O_3_	2.29(2)	3.2
O_4_	2.42(1)	2.2	O_4_	2.40(2)	1.5	S_1_	2.67(3)	0.5
Fe_1_	3.22(1)	2	Fe_1_	3.21(1)	2	Fe_1_	3.21(5)	0.5
Fe_2_	3.44(2)	2	Fe_2_	3.44(1)	2	Fe_2_	3.44(6)	0.5
Fe_3_	3.65(1)	3	Fe_3_	3.64(1)	3			
Fe_4_	4.71(3)	1	Fe_4_	4.70(3)	1			
Fe_5_	5.32(2)	2	Fe_5_	5.30(2)	3			
Fe_6_	5.63(3)	2	Fe_6_	5.61(2)	4			
Fe_7_	5.90(4)	2	Fe_7_	5.88(3)	3			

a Standard taken from a previous study.^23^

After 1 day of sulfidation, the best fit model contains 3 O backscatterers at 2.24 Å, 3 O backscatterers at 2.38 Å, 0.5 Fe backscatterers at 3.29 and 3.47 Å, and 1 U backscatterer at 3.71 Å, suggesting a complex mixture of uranium coordination environments and oxidation states (Table 2). First, the 3 O backscatterers at 2.38 Å and the 1 U backscatterer at 3.71 Å suggest some UO_2_ formation, with the low U–U coordination number, indicating a poorly crystalline UO_2_ phase (cf. crystalline UO_2_ with 8 O at 2.37 Å and 12 U at 3.87 Å).^62^ However, the 3 O backscatterers at 2.24 Å may suggest the presence of a U_4_O_9_ (U(V)_2_U(IV)_2_O_9_) or U_3_O_7_ phase (cf. U_4_O_9_, U–O at 2.25 Å and U–U at 3.87 Å; U_3_O_7_, U–O ∼2.3 Å and U–U ∼3.9 Å).^70−72^ This further supports the hypothesis that a mixed-valence U oxide species is present, in addition to poorly crystalline UO_2_, providing a possible explanation for the U(IV) and U(V) components observed in the M_IV_-edge HERFD-XANES spectra (Figure 2). In addition, the presence of 2 U–Fe shells at 3.29 and 3.47 Å indicate that a residual U–goethite phase (either U(V) or U(VI)) is retained in the system after 1 day of sulfidation. Interestingly, a modest but persistent U(VI) component was still identified by M_IV_-edge HERFD-XANES after 9 months (Figure 2), and goethite particles were identified by TEM imaging after 7 months, suggesting that a fraction of U(VI)–goethite is retained long-term in the system. Indeed, U(VI)–goethite reportedly undergoes partial reduction to a mixed U(V)/U(VI)–goethite species on reaction with aqueous Fe(II).^23^ Therefore, given that the coordination environment for both U(V)– and U(VI)–goethite includes O backscatterers at approximately 2.2 and 2.4 Å (cf. 2.24 and 2.38 Å after 1 day of sulfidation), and given the highly reducing conditions in the sulfidic system, both U(V)– and U(VI)–goethite incorporated species may contribute to the persistent U(V) and U(VI) components of the M_IV_-edge HERFD-XANES spectra (Figure 2). Consequently, the presence of U(V) in the system may be due to either a partially reduced U(VI/V)–goethite species (electron transfer from aqueous Fe(II) and/or aqueous HS^–^) and/or the formation of mixed-valence U oxides (e.g., U_4_O_9_) formed upon reduction of U(VI) by FeS.

**Table 2:** Details of the EXAFS Fits for 1 Day, 1 Week, 3 Months, and 6 Months of U(VI)–Goethite Sulfidation Samples (Full Details in the Supporting Information, Table S2)

1 day			1 week			3 months			6 months		
path	*R* (Å)	CN	path	*R* (Å)	CN	path	*R* (Å)	CN	path	*R* (Å)	CN
O_1_	2.24(2)	3	O_1_	2.26(2)	3	O_1_	2.31(1)	4	O_1_	2.32(1)	5
O_2_	2.38(2)	3	O_2_	2.40(2)	3	O_2_	2.46(2)	2	O_2_	2.48(2)	2
Fe_1_	3.29(3)	0.5	U_1_	3.82(3)	1	U_1_	3.86(1)	5	U_1_	3.86(1)	8
Fe_2_	3.47(4)	0.5				O_3_	4.42(2)	8	O_3_	4.44(1)	14
U_1_	3.71(3)	1									

By 1 week of sulfidation, the best fit model was 3 O backscatterers at 2.26 Å, 3 O backscatterers at 2.40 Å, and 1 U backscatterer at 3.82 Å, with a notable elongation in the U–U interatomic distance from 3.71 to 3.82 Å, possibly indicating a more crystalline UO_2_ phase.^73^ After 3 months, the best fit model contained 4 O backscatterers at 2.31 Å, 2 O backscatterers at 2.46 Å, 5 U backscatterers at 3.86 Å, and 8 distal O backscatterers at 4.42 Å. This suggests that at 3 months, U is still present as a complex mixture of nanocrystalline UO_2,_ mixed valence U oxide and/or U–goethite. Specifically, nanocrystalline UO_2_ contains a U(IV) coordinated by 8 O ions at 2.37 Å, which is an approximate mid-point between the fitted O backscatterers at 2.31 and 2.46 Å. In addition, the 5 U backscatterers (3.86 Å) and 8 distal O backscatterers (4.42 Å) correlate well with nanocrystalline UO_2_ (U(IV)O_2_ = 12 U at 3.87 Å, 24 O at 4.53 Å),^62^ which supports the progressive U(IV) formation observed in both L_III_-edge XANES (Figure S16) and M_IV_-edge HERFD-XANES (Figure 2). By 6 months, the best-fit EXAFS model shows a marked increase in the crystallinity of the UO_2_, with 5 O backscatterers at 2.32 Å, 2 O backscatterers at 2.48 Å, 8 U backscatterers at 3.86 Å, and 14 distal O backscatterers at 4.44 Å. Therefore, overall, U is partitioned to the solid phase during sulfidation and is mainly retained as nanocrystalline UO_2_, with a minor amount of mixed valence U oxide and/or U–goethite evident after several months of reaction.

### Reoxidation Experiment

Given fluctuating redox conditions in the environment, to better understand the long-term fate of U-incorporated iron (oxyhydr)oxide phases, a controlled reoxidation experiment was performed (pH 7, 5% DO). A chemostat system was utilized to maintain the pH (via acid/base additions) and DO content (via air/N_2_ flow) and to monitor the redox potential during reoxidation.

**Figure 4:**
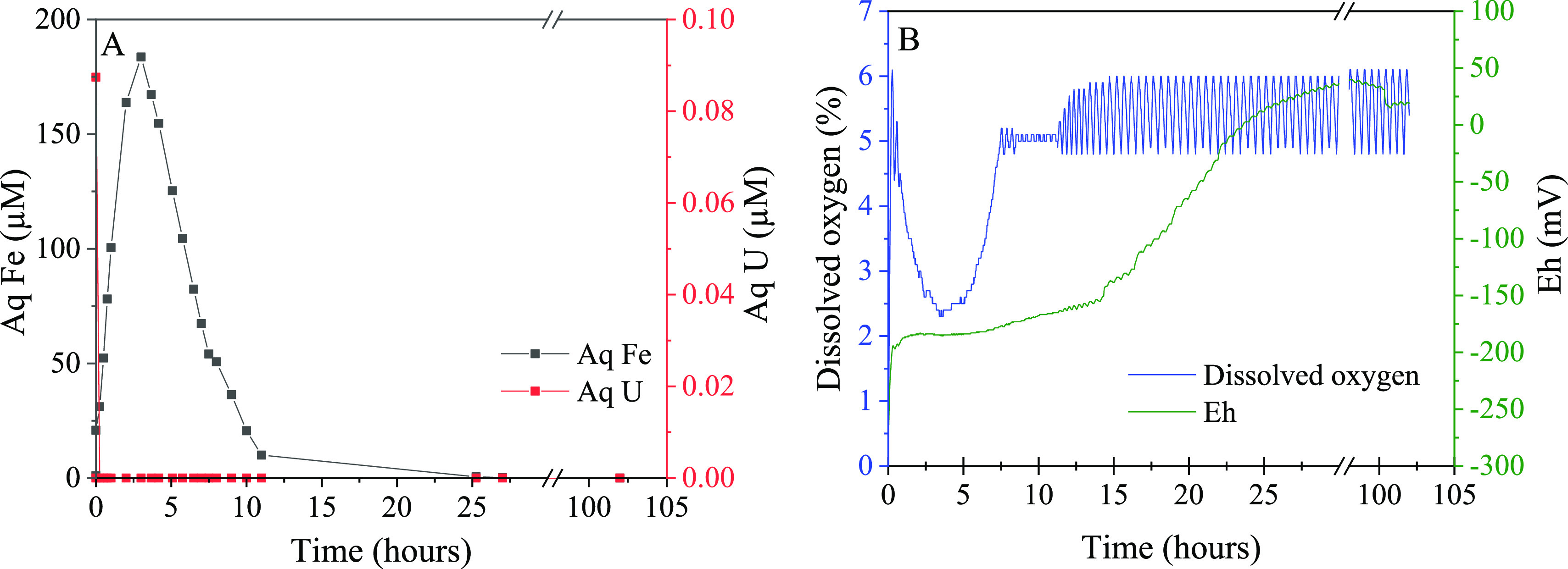
Solution data for the controlled reoxidation experiment. (A) Measured aqueous species (U analyses < 0.01 μM are considered below detection); (B) redox data.

First, despite a constant flow of laboratory air, DO levels initially decreased over the first 5 h to a minimum of 2.5%, followed by a gradual increase up to the set-point of 5% DO (7.5 h; Figure 4B). During this time, the redox potential (Eh) remained relatively low, rising only 10 mV between 1 and 7.5 h (−187 to −177 mV; Figure 4B). Between 7.5 and 11.5 h, minimal air and/or nitrogen were needed to maintain a DO level of 5% (Figures S5 and S6), and there was a gradual increase in Eh of 14 mV over the 4 h (−163 mV, 11.5 h). However, Eh then rapidly increased, with an Eh of +2.7 mV by 24 h. Moreover, from 11.5 h onwards, a repeated intermittent flow of nitrogen was needed to maintain a 5% DO level with minimal laboratory air (Figure S6), suggesting that the redox buffering capacity of the experiment was limited from 11.5 h onward.

After 4 days of oxygen ingress (at 5% DO), XRD revealed that a mixture of lepidocrocite (γ-FeOOH), goethite (α-FeOOH), and elemental sulfur had formed in the system (Figure S14). This was corroborated in TEM images, which showed clusters of goethite rods and lepidocrocite laths (100–200 nm, Figures S12 and S13).^39^ Therefore, the observed geochemical behavior (i.e., DO and Eh) was likely due to the redox buffering effect of FeS, which has been shown to oxidize by the following equation^55^
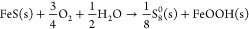


In the past work on synthetic nanocrystalline FeS mixed with uraninite, FeS was shown to preferentially oxidize before U(IV)O_2_, thereby “buffering” U(IV)O_2_ reoxidation.^55^ In the current study, the oxidation of FeS was indicated by the release of aqueous Fe, which reached a maximum concentration of 184 μM after 3 h (Figure 4A). This was followed by a steady decrease in aqueous Fe to a minimum of 10 μM at 11 h, likely due to the oxidation of released Fe(II) and subsequent formation of iron (oxyhydr)oxide phases (FeOOH). Therefore, the DO behavior over the first 11 h was likely controlled by the kinetics of FeS oxidation, which consequently buffered any rapid increase in the redox potential of the system. Furthermore, on the introduction of air to the system, although there was an immediate release of 0.09 μM aqueous U (∼2% of total U at 1 min), from 15 min onward, there was no aqueous U above the detection limit (Figure 4A). This rapid U release suggests that, despite the reported oxygen scavenging behavior of synthetic nanocrystalline FeS, there may be simultaneous oxidation of the U(IV)O_2_ and FeS in the chemostat experimental system. Any released U(VI) will have then been immediately adsorbed and/or incorporated onto the newly formed iron (oxyhydr)oxide (FeOOH) phase.^23,74^

To assess the association of U with the iron (oxyhydr)oxide species (e.g., adsorbed or incorporated), surface-bound U is often removed using techniques such as a bicarbonate extraction (for adsorbed U(VI)/non-crystalline U(IV))^18,75^ or a sequential acid extraction (for adsorbed U(VI)/U(IV)O_2_).^15,20,23^ Here, following 4 days of reoxidation, 65% U was found to be bicarbonate-extractable, consistent with 62% for a 0.1 M HCl extraction (Figure S15). Therefore, after 4 days of reoxidation, approximately 35% U appears to be resistant to bicarbonate or acid leaching and is potentially incorporated within an iron (oxyhydr)oxide (i.e., goethite or lepidocrocite), with 65% U likely adsorbed.

To further probe the speciation of the U-associated iron (oxyhydr)oxide species, U L_III_-edge XANES were collected at selected timepoints during reoxidation (Figure S19A–C). The collected spectra reveal a small but progressive increase in the XANES edge position over the first 11 h of reaction (∼0.6 eV), indicating partial U oxidation (Figure S19B). By 4 days, there was a significant increase in the XANES edge position (∼2.7 eV), suggesting that oxidation to U(VI) was complete. Overall, this confirms that simultaneous reoxidation of U(IV)O_2_ and FeS occurred. During FeS oxidation (approximately 11 h), a fraction of U was oxidized to U(VI); this U(VI) may have then been incorporated into the growing iron (oxyhydr)oxide phases (up to ∼35% U incorporation from the bicarbonate and acid leaching). The majority of U reoxidation then occurred on depletion of FeS and the associated redox buffering to form U(VI)-adsorbed iron (oxyhydr)oxides as the predominant species. In addition, XANES were collected on a sample after acid extraction of surface-bound (i.e., adsorbed) U; the edge position of the U L_III_-edge XANES decreased in energy, suggesting that a more reduced U species was present in the acid-leached sample, where adsorbed U(VI) had been preferentially removed (Figure S19C). This suggests that although the adsorbed fraction (65% U) consists of a U(VI) species, the incorporated fraction (35% U) has a lower overall oxidation state, potentially with a U(V) component. Interestingly, during the sulfidation of U(VI)-incorporated goethite, there was evidence for U(V)-incorporated goethite formation (Figure 2). As previous studies have found the U in U(V)-incorporated iron (oxyhydr)oxides to be resistant to reoxidation,^17,21^ the presence of a recalcitrant U(V)-incorporated goethite component after reoxidation further supports the hypothesis that U(V)-incorporated goethite was formed during U(VI)–goethite sulfidation.

The U behavior and speciation during reoxidation were further explored using U L_III_-edge EXAFS. Firstly, EXAFS best fits for 3–5 and 7.5–11 h are very similar, with 2 O backscatterers at 2.19–2.21 Å, 4 O backscatterers at 2.37 Å and 3 U backscatterers at 3.82–3.85 Å (Table S3, Supporting Information). Therefore, although there was a slight increase in the energy of the XANES edge position, which indicates U oxidation (Figure S19B), there was still significant UO_2_, and possibly mixed valence U oxides (e.g., U_4_O_9_), retained after 11 h of reoxidation. By 4 days of air reoxidation, the EXAFS best fit contained 2 O at 1.81 Å, 2 O at 2.29 Å, 2 O at 2.44 Å and 0.5 Fe at 3.44 Å, confirming that U(VI) was predominantly in a uranyl coordination (Table S3). Therefore, the dominant U speciation was likely an adsorbed uranyl goethite/lepidocrocite phase, which is consistent with the chemical extraction results. Given that U(VI)–goethite has undergone a substantial dissolution and recrystallization process (i.e., sulfidation), incorporated U(VI) was expected to be transferred into a more labile phase (65% of U has transformed from recalcitrant to bicarbonate extractable). Therefore, finding a significant fraction of U (35%) still retained within the iron (oxyhydr)oxide following redox cycling, despite the substantial reductive dissolution of U(VI)–goethite, is a significant result. This suggests that under ambient environmental conditions, even when subject to dissolution and transformation reactions (i.e., sulfidation followed by oxidation), iron (oxyhydr)oxides will hinder U mobility (i.e., 35% incorporated U(VI)/(V)). The newly formed labile fraction (i.e., 65% adsorbed U(VI)) has the potential to be remobilized (e.g., by complexation with carbonate ligands) or may become re-incorporated during further recrystallization of iron (oxyhydr)oxides during subsequent redox cycling.^16,39^

## Environmental Implications

Using a combination of XAS and geochemical analysis, the behavior and speciation of U during the sulfidation and subsequent reoxidation of U(VI)-incorporated goethite have been explored. Initially, remobilization of a transient aqueous U species was observed during sulfidation, with EXAFS analysis indicating the formation of a U(VI)–persulfide species, which was partially adsorbed to the solid phase at 4 h. However, in the long term, U was largely retained in the solid phase as nano-crystalline U(IV)O_2+*x*_. Interestingly, TEM images indicated that a modest fraction of residual U-incorporated goethite was also retained after several months. During controlled air reoxidation, mackinawite then transformed to a mixed goethite/lepidocrocite phase, which contained adsorbed U(VI) (approximately 65%) and U(V/VI)-incorporated (approximately 35%) iron (oxyhydr)oxide species. In contaminated land and waste disposal scenarios, a complex combination of biotic and abiotic interactions will occur, where sulfate-reducing bacteria will produce aqueous sulfide in-situ and microorganisms can also directly reduce both U(VI) and iron (oxyhydr)oxides.^1,2^ In addition, sulfide is present in some groundwaters^56^ and may be transported in the sub-surface. This study focuses on further understanding the abiotic interactions that may occur between aqueous sulfide (at environmentally relevant concentrations^56^) and U-associated iron (oxyhydr)oxides. While reaction with aqueous sulfide may initially release a modest fraction of aqueous U (e.g., 2.5%), the aqueous U species is short-lived and rapidly reduced to solid-phase UO_2+*x*_. In addition, despite excess aqueous sulfide, a fraction of U(V/VI)-incorporated goethite was resistant to reductive dissolution, and partial reincorporation of U with goethite/lepidocrocite may have occurred during reoxidation. Consequently, although U is released from the goethite structure during sulfidation, under the environmental conditions (i.e., sulfate-reducing conditions followed by oxygen ingress), a U-incorporated goethite component may persist in the sub-surface in the long term.

## Supporting Information Available

Additional information on the geochemical analyses, M_IV_-edge HERFD-XANES, and U L_III_-edge XAS data (PDF)
