# Supplementary material for: Sulfidation and Reoxidation of U(VI)-Incorporated Goethite: Implications for U Retention during Sub-Surface Redox Cycling
Source: Environ Sci Technol. 2022 Nov 30;56(24):17643–52. doi: 10.1021/acs.est.2c05314 (PMC9775214; doi:10.1021/acs.est.2c05314)
Supplement: Supplementary file 1 — es2c05314_si_001.pdf [file es2c05314_si_001.pdf]

Supporting Information

Sulfidation and reoxidation of U(VI)-incorporated goethite: implications for U retention during sub-surface redox cycling

Olwen Stagg,[1‡] Katherine Morris,[1] Luke Thomas Townsend,[1,†] Kristina O. Kvashnina,[2,3]*

Michael L. Baker,[4,5] Ryan L. Dempsey,[4] Liam Abrahamsen-Mills[6] and Samuel Shaw[1]*

[1]Research Centre for Radwaste Disposal and Williamson Research Centre for Molecular Environmental Science, Department of Earth and Environmental Sciences, The University of Manchester, Manchester, M13 9PL, U.K.

[2]The Rossendorf Beamline at ESRF – The European Synchrotron, CS40220, 38043 Grenoble Cedex 9, France.

[3]Helmholtz Zentrum Dresden Rossendorf (HZDR), Institute of Resource Ecology, 01314, Dresden, Germany

[4]Department of Chemistry, The University of Manchester, Manchester, M13 9PL.

[5]The University of Manchester at Harwell, The University of Manchester, Diamond Light Source, Harwell Campus, OX11 0DE, U.K.

[6]National Nuclear Laboratory, Warrington, Cheshire, WA3 6AE, U.K.

*Corresponding author email:
sam.shaw@manchester.ac.uk and olwen@wustl.edu

Pages: 21

Figures: 20

Tables: 3

This document provides supporting information for this paper, which includes the presentation of additional geochemical analyses, XRD and TEM images, and XAS data. To obtain physical access to the data files please contact the corresponding author.

Contents

Section S1: Geochemical Analyses 3

1.1 Sulfidation of U(VI)-goethite 3

1.2 Re-oxidation of sulfidised U(VI)-goethite 4

Section S2: Solid phase analysis 6

2.1 Sulfidation of U(VI)-goethite 6

2.2 Re-oxidation of sulfidised U(VI)-goethite 8

Section S3: XAS analysis 11

3.1 Sulfidation of U(VI)-goethite 12

3.2 Re-oxidation of sulfidised U(VI)-goethite 18

Section S1: Geochemical Analyses

1.1 Sulfidation of U(VI)-goethite

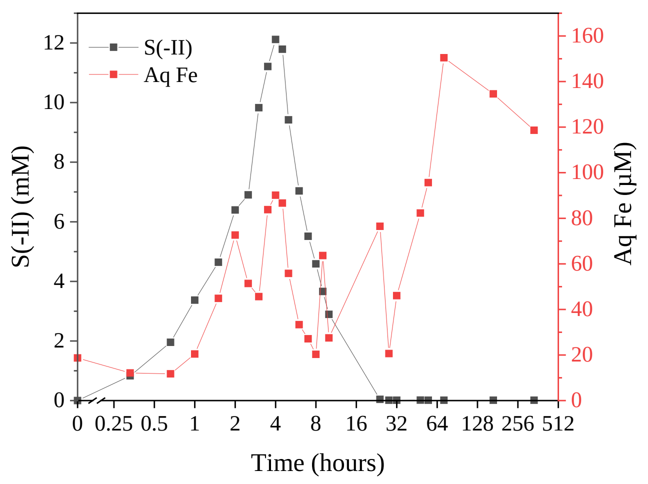

Figure S1. Aqueous Fe and S(-II) during the sulfidation of U(VI)-incorporated goethite. The *x*-axis is shown as $\log_2$ after 0 hours.

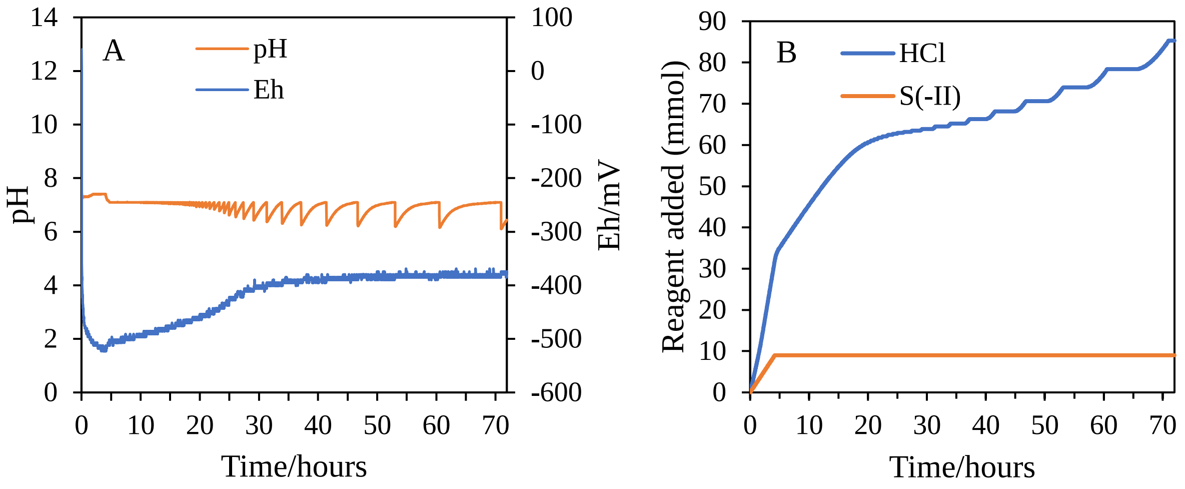

Figure S2. Geochemical data (A) and reagent additions (B) measured during the sulfidation of U(VI)-incorporated goethite.

1.2 Re-oxidation of sulfidised U(VI)-goethite

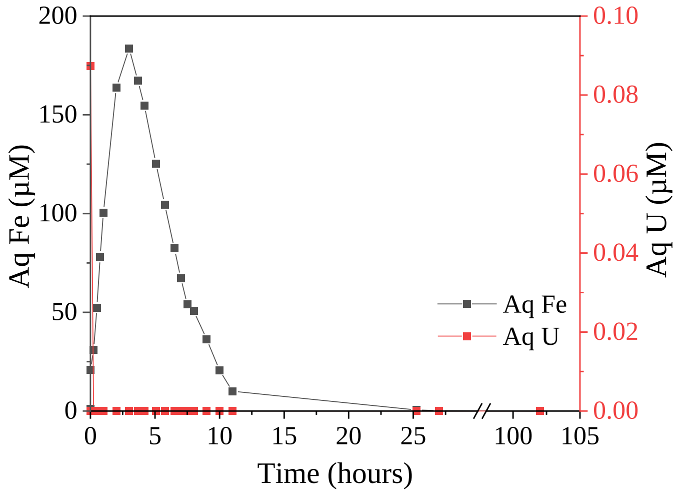

Figure S3. Aqueous Fe and U during the re-oxidation of sulfidised U(VI)-goethite.

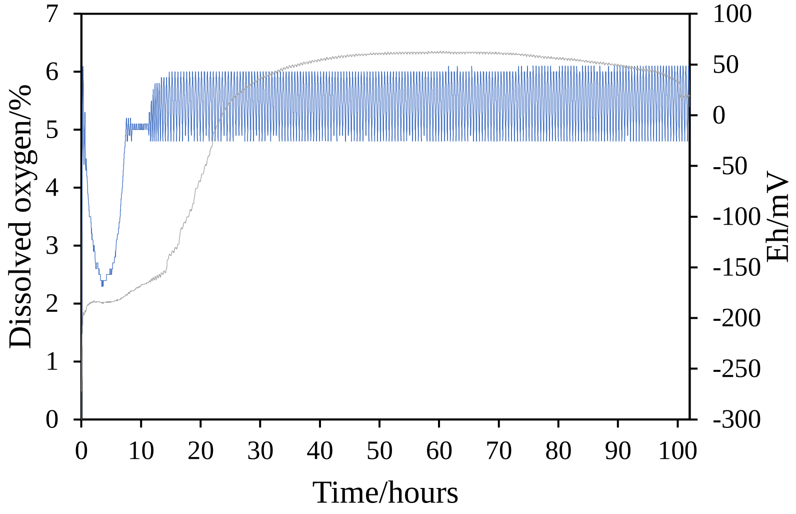

Figure S4. Measurements of dissolved oxygen and redox potential during the re-oxidation of sulfidised U(VI)-goethite.

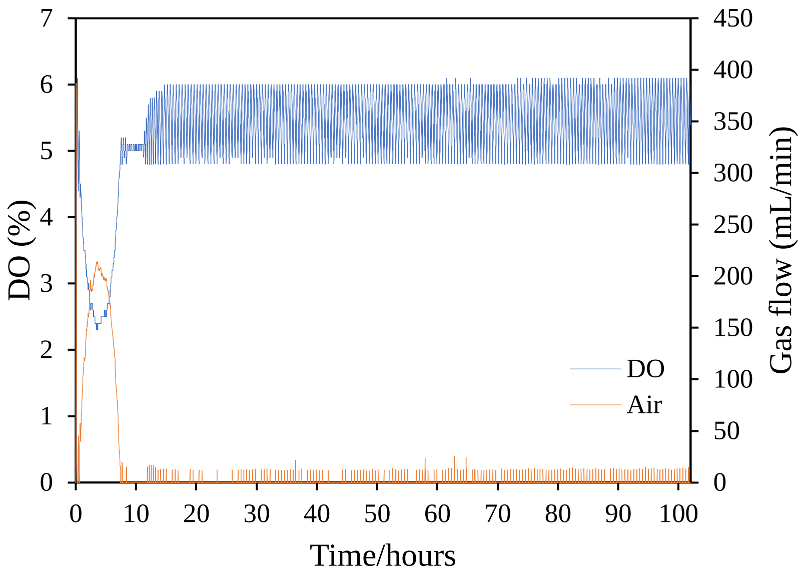

Figure S5. Measurements of dissolved oxygen and laboratory air flow during the re-oxidation of sulfidised U(VI)-goethite.

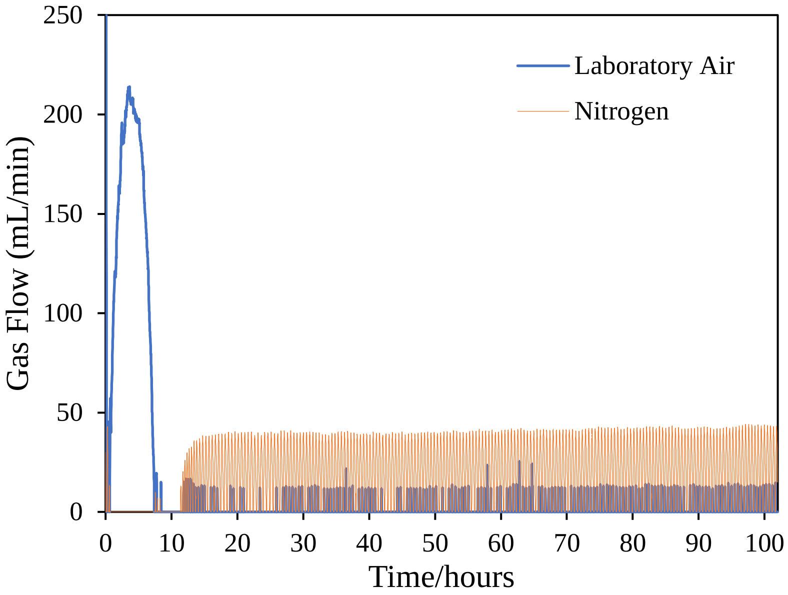

Figure S6. The gas flow rate of laboratory air and nitrogen during the re-oxidation of sulfidised U(VI)-goethite.

Section S2: Solid phase analysis

2.1 Sulfidation of U(VI)-goethite

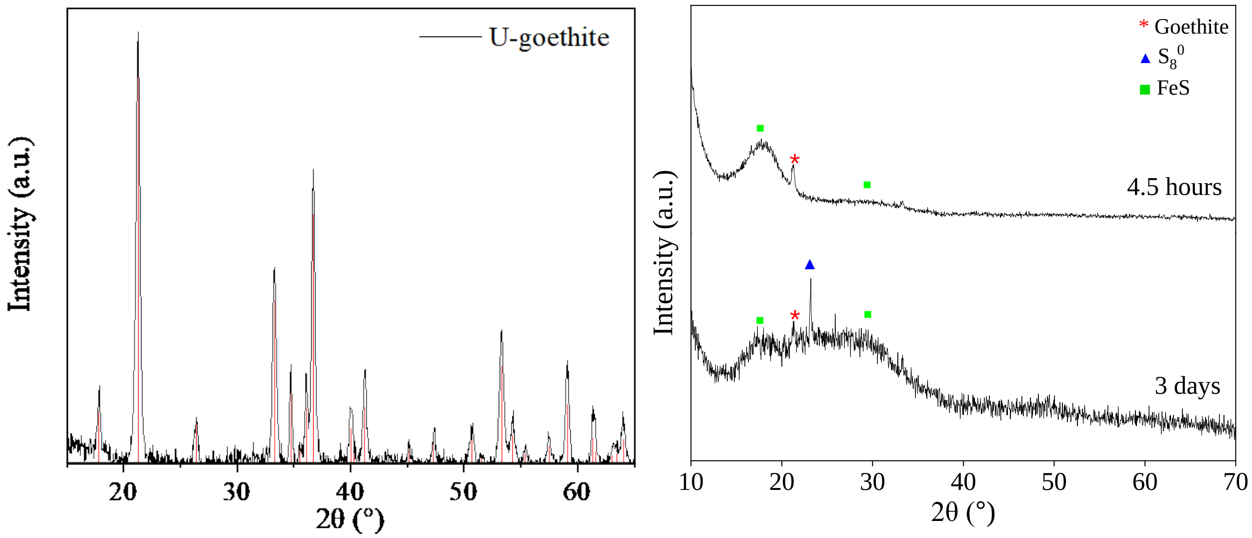

Figure S7. XRD pattern of (left) U(VI)-incorporated goethite starting material, with red lines displaying goethite reflections; (right) 4.5 hours and 3 days U(VI)-goethite sulfidation, revealing the significant depletion in goethite, and ingress of elemental sulfur (S_0^8) and an amorphous FeS phase.

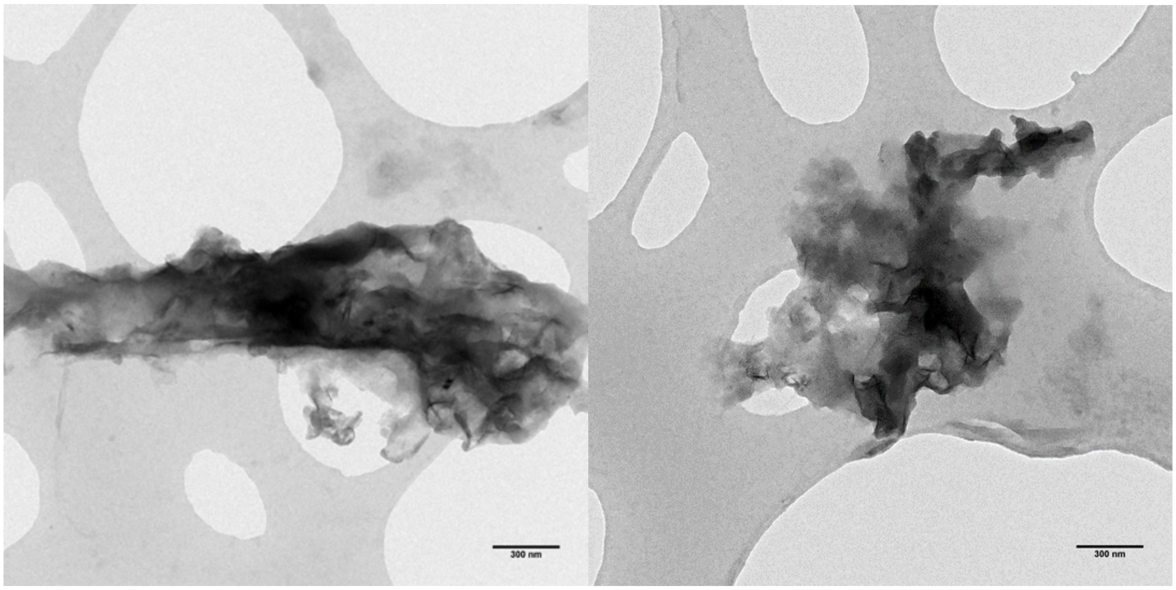

Figure S8. TEM images of FeS, formed after 1 day of U(VI)-goethite sulfidation.

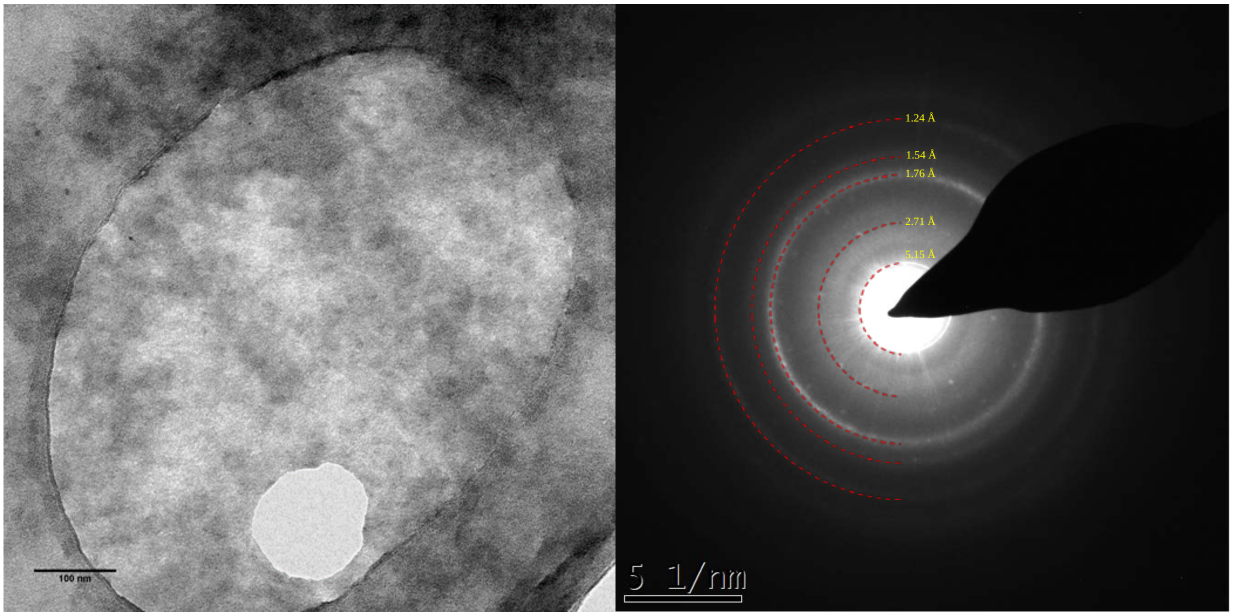

Figure S9. TEM image of amorphous 7 month sulfidation phase, with associated SAED pattern displaying d-spacings matching FeS.

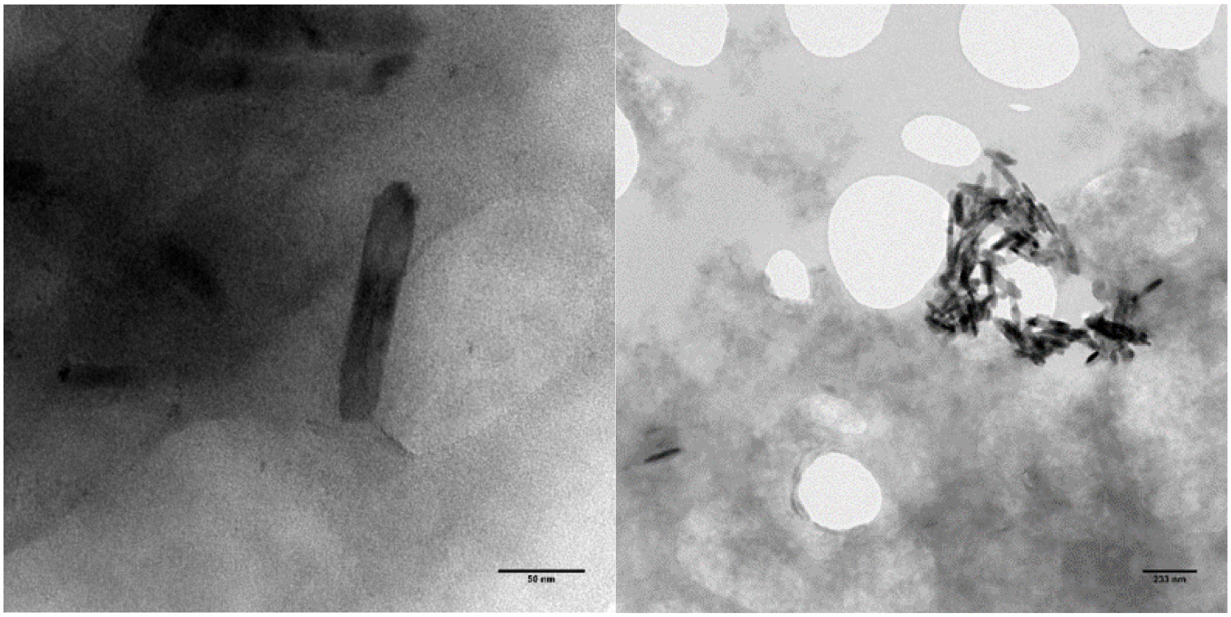

Figure S10. TEM images of rod-like morphologies, found after 7 months of U(VI)-goethite sulfidation.

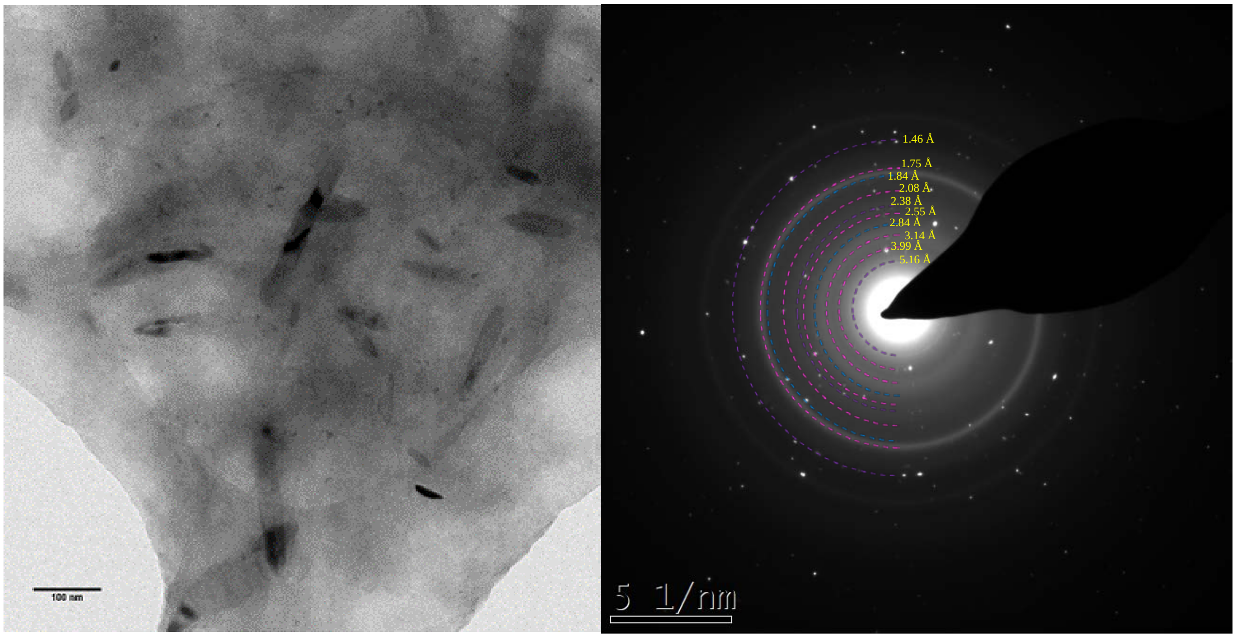

Figure S11. TEM image of a cluster of rod-like particles in 7 months U(VI)-goethite sulfidation sample, with associated SAED pattern displaying rings form both FeS (blue/purple) and goethite (pink/purple).

2.2 Re-oxidation of sulfidised U(VI)-goethite

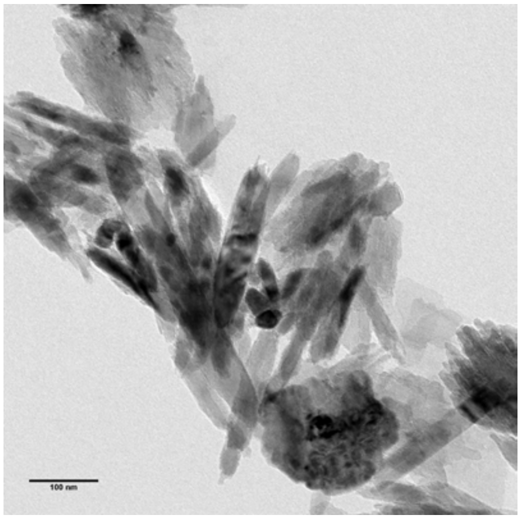

Figure S12. TEM image of goethite/lepidocrocite rods/laths, formed after 4 days re-oxidation.

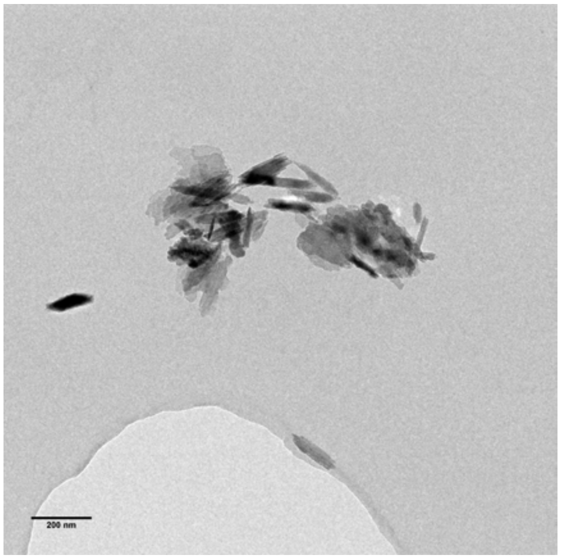

Figure S13. TEM image of goethite/lepidocrocite rods/laths, formed after 4 days re-oxidation.

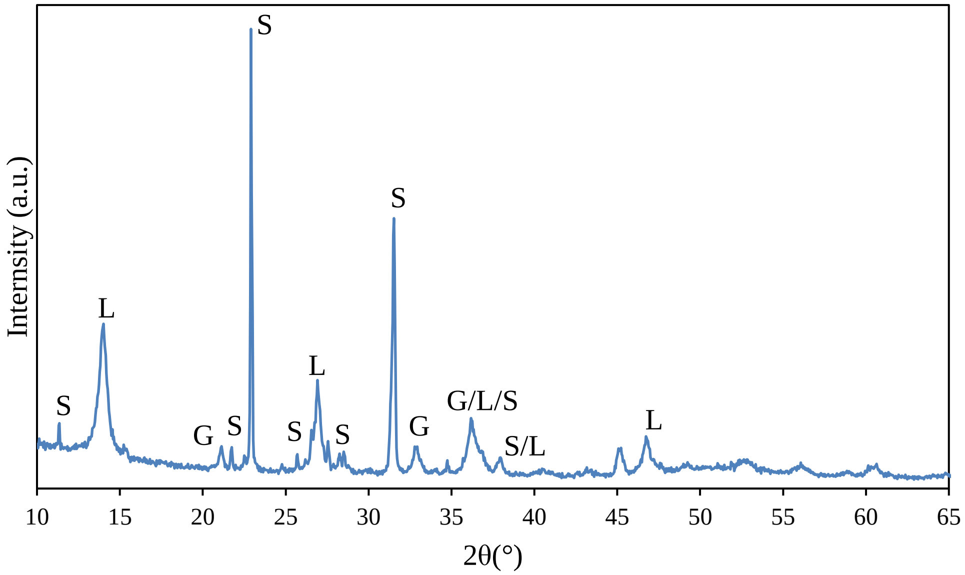

Figure S14. X-ray diffraction pattern of the 4-day re-oxidation timepoint, with prominent peaks labelled as S (sulfur), G (goethite) and/or L (lepidocrocite).

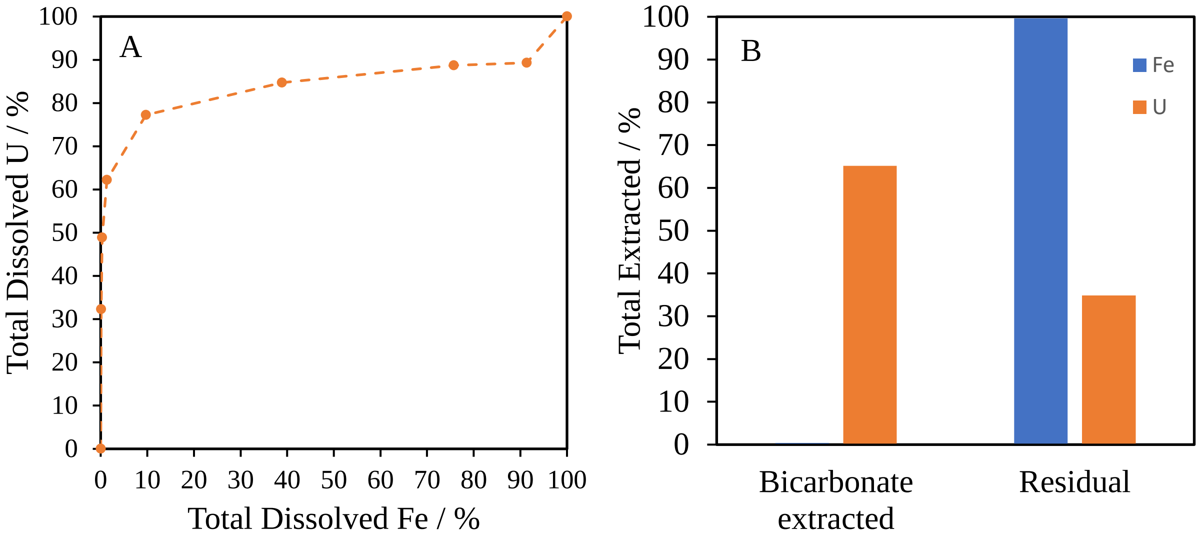

Figure S15. Acid dissolution profile (A) and bicarbonate extraction (B) for 4 day re-oxidation sample.

Section S3: XAS analysis

For XAS analysis, solid samples were obtained by filtration (nylon membrane filter, 0.22 μm), then stored and transported at -80 °C under anoxic conditions to the Diamond Light Source (UK) for analysis on either the I20-scanning or B18 beamline. XAS spectra were collected at the U L_{III}-edge in fluorescence mode at 80 K, using 64-element (I20) and 36-element (B18) Ge detectors. The resulting data was processed using the Demeter software package, using Athena and Artemis with FEFF6.[1] For HERFD-XANES, select solid filtrate samples were transported frozen and under anoxic conditions to the European Synchrotron Radiation Facility (ESRF) in Grenoble. Measurements were performed at beamline BM20,[2] and the incident energy was selected using the <111> reflection from a double Si crystal monochromator. X-ray absorption near edge structure (XANES) spectra were measured in HERFD mode using an X-ray emission spectrometer,[3] and the sample, analyzer crystal and photon detector (silicon drift detector, Ketek) were arranged in a vertical Rowland geometry. The size of the beam at the sample, which defines the total energy resolution of the HERFD-XANES data, was 35 μm in a vertical direction. U M_{IV}-edge HERFD-XANES spectra were collected using the U Mβ emission line (~3337 eV),[4,5] under cryo conditions with Oxford Cryostream (800 series) at 50K. Subsequent data analysis was performed using the ITFA software package.[6] Interestingly, recent evidence suggests that uncertainties might be present in M_{IV}-edge analyses,[7,8] thus the values are presented here with a semi-quantitative interpretation.

ITFA Analysis

ITFA analysis[6] was performed on U M_{IV}-edge HERFD-XANES spectra (U(VI)-goethite sulfidation), to gain insight into the proportion of U(VI), U(V) and U(IV) in samples. Firstly, a principal component analysis was run on the samples, along with a variety of U(VI), U(V), and U(IV) containing standards. This revealed that only 3 significant eigenvectors contributed to the spectra, confirming that only 3 components were present (i.e. U(VI), U(V) and U(IV)). The known standards were then used to fit the sample spectra, and determine the concentration of U(VI), U(V), and U(IV) in the samples. For further accuracy, multiple ITFA analyses were conducted (5 ITFA runs) using a different combination of standards (e.g. U(VI)-goethite, U_3O_7 and U_3O_8 vs U(VI)-goethite, U(V)-goethite and $U(IV)O_2$). This enabled an average percentage concentration to be determined from the collection of ITFA runs, and from this a standard error was calculated. Further details of these ITFA analyses are shown below, in Table S1 and Figure S17.

3.1 Sulfidation of U(VI)-goethite

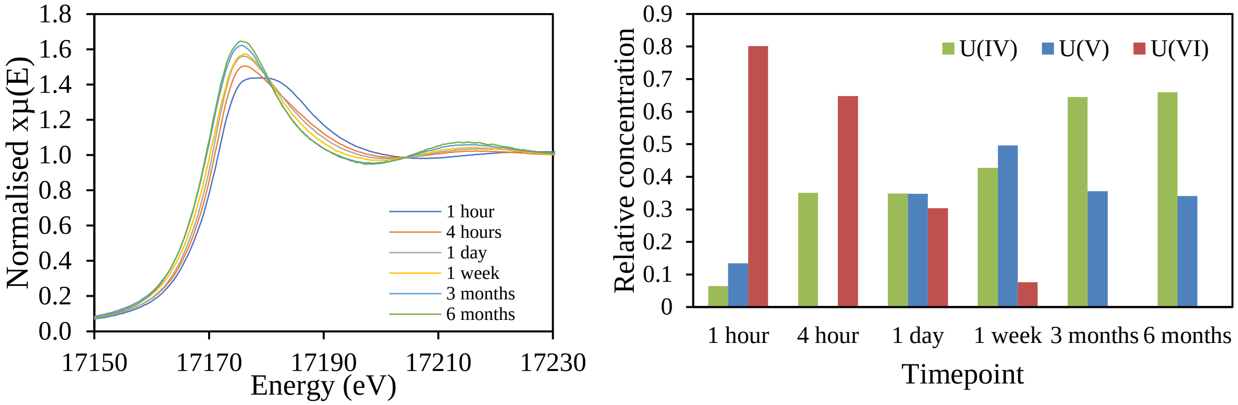

Figure S16. U L_{III}-edge XANES (left) and linear combination fitting results (right) for U(VI)-goethite sulfidation samples. Linear combination fitting performed using acid-washed U(VI)-goethite, U(V)-goethite and $U(IV)O_2$ standards

Table S1. Details of ITFA[6] fitting results for collected U M_{IV}-edge HERFD-XANES spectra (U(VI)-goethite sulfidation), including standards used and calculated errors.

Run	Standards	ITFA Result								
		1 hour			4 hour			9 months		
		U(VI)	U(V)	U(IV)	U(VI)	U(V)	U(IV)	U(VI)	U(V)	U(IV)
**A**	U(VI)-goethite, U_3O_7, U_3O_8, U_4O_9, UO_2, UO_3	98	0	4	58	7	35	15	8	77
**B**	U(VI)-goethite, U_3O_7, U_3O_8	90	8	2	54	14	32	18	12	70
**C**	U(VI)-goethite, U(V)-goethite, UO_2	92	6	2	38	33	28	5	21	73
**D**	U(VI)-goethite, U_3O_7, UO_2	91	7	2	50	17	34	11	12	77
**E**	U(VI)-goethite, U_3O_8, UO_2	90	8	3	49	16	35	7	16	77

	Average ITFA Result								
	1 hour			4 hour			9 months		
	U(VI)	U(V)	U(IV)	U(VI)	U(V)	U(IV)	U(VI)	U(V)	U(IV)
Relative Concentration (%)	92	5	3	50	17	33	11	14	75
Error (+/- SE)	1.3	1.3	0.4	3.0	3.8	1.2	2.2	2.0	1.3

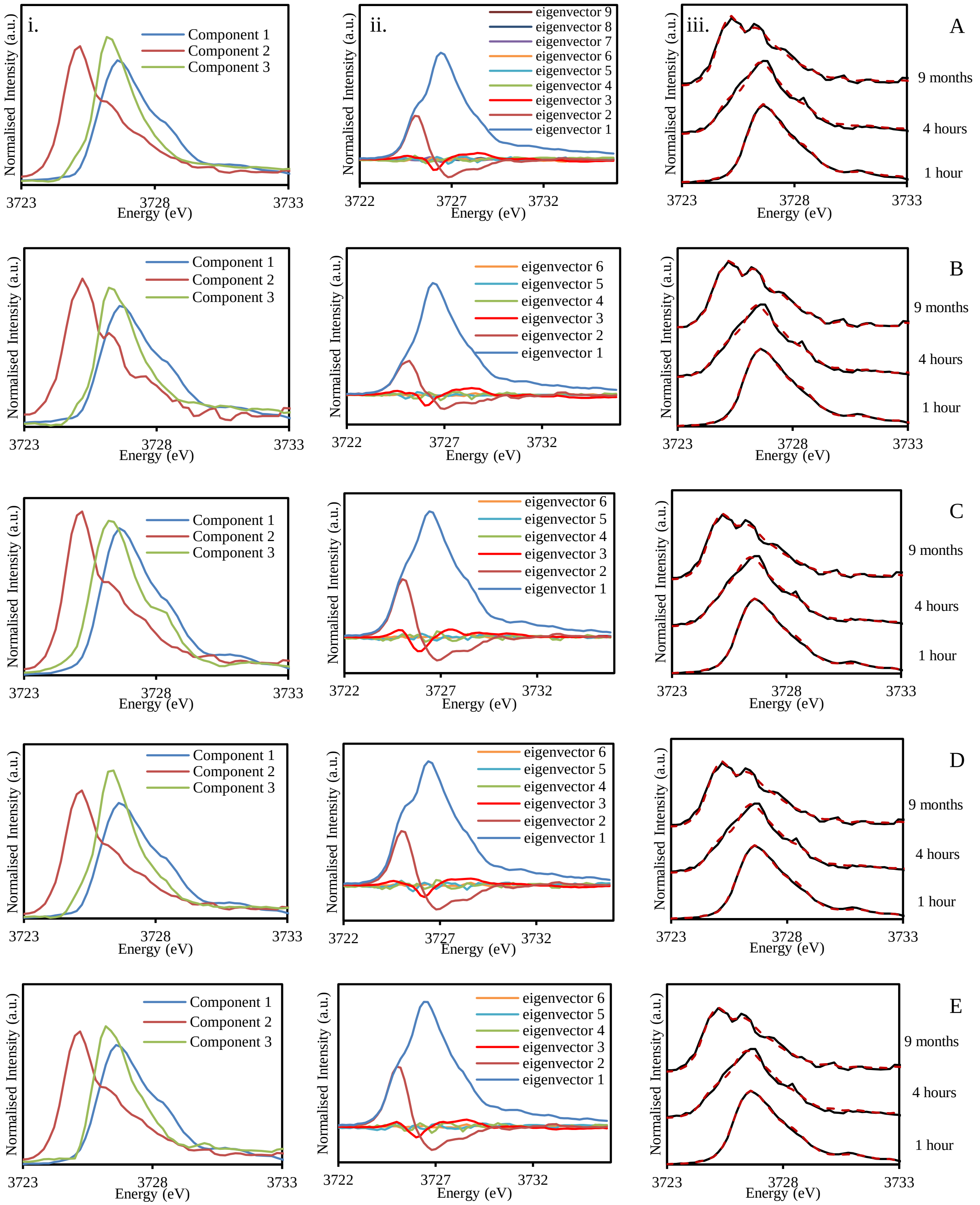

Figure S17. ITFA results for runs A-E (Table S1). (i) component spectra produced by ITFA programme; (ii) ITFA-extracted eigenvector contributions, which indicate that only eigenvectors 1-3 contribute significantly to the data and therefore only 3 components are needed to reproduce sample spectra; (iii) sample (solid black lines) and reproduced (dashed red lines) spectra.

Table S2. Details of EXAFS best fit parameters from sulfidised U(VI)-goethite. CN denotes coordination number; R denotes the atomic distance; σ^2 denotes the Debye-Waller factor; α denotes the statistical significance of each shell from the F-test, determined from whether the fit was significantly worsened on removal of an individual Fe shell; E_0 denotes the shift in energy from the calculated Fermi level; S_0^2 denotes the amplitude factor; χ_v^2 denotes the reduced χ squared value; R-factor describes the "goodness of fit"; V is the number of variables; NIDP is the number of independent points. All CN and $S0^2$ values are fixed. [†]Indicates the two parameters were linked in a given fit. [a]R range = 1.15-6; [b]R range = 1.15-4; [c]R range = 1.15–4.2; [d]R range = 1.15-4.3; [e]K range = 3-12.5; [f]K range = 3-13; [g]K range = 3-12; [h]K range = 3-10.5.

Sample	Path	CN	R (Å)	σ^2 (Å^2)	α (%)	E_0 (eV)	S_0^2	χ_v^2	R-factor	V/NIDP
**1 hour[a,e]**	U-O_1	1	1.879 ± 0.012	0.006 ± 0.001	99.8					
	U-O_2	1	2.056 ± 0.028	0.006 ± 0.002[†]	98.8					
	U-O_3	2.5	2.229 ± 0.015	0.006 ± 0.002[†]	100					
	U-O_4	1.5	2.404 ± 0.020	0.007 ± 0.003	100					
	U-Fe_1	2	3.208 ± 0.008	0.006 ± 0.001[(2†)]	100					
	U-Fe_2	2	3.439 ± 0.012	0.006 ± 0.001[(2†)]	100	6.11	0.9	40.74	0.011	19/28.98
	U-Fe_3	3	3.644 ± 0.010	0.007 ± 0.0005	100					
	U-Fe_4	1	4.696 ± 0.028	0.007 ± 0.002[(3†)]	99.9					
	U-Fe_5	3	5.304 ± 0.017	0.007 ± 0.002[(3†)]	100					
	U-Fe_6	4	5.608 ± 0.020	0.009 ± 0.002[(4†)]	99.9					
	U-Fe_7	3	5.882 ± 0.033	0.009 ± 0.002[(4†)]	97.2					

**4 hour[b,f]**	U-O_1	1	1.853 ± 0.012	0.005 ± 0.001[†]	100	-1.381	0.9	86.79	0.0185	11/17.88
	U-O_2	1.8	2.113 ± 0.020	0.006 ± 0.002	99.9					
	U-O_3	3.2	2.289 ± 0.015	0.005 ± 0.001[†]	100					
	U-S	0.5	2.668 ± 0.029	0.005 ± 0.002	100					
	U-Fe_1	0.5	3.211 ± 0.047	0.009 ± 0.005[(2†)]	91.8					
	U-Fe_2	0.5	3.438 ± 0.059	0.009 ± 0.005[(2†)]	92.8					
**1 day[b,g]**	U-O_1	3	2.242 ± 0.017	0.009 ± 0.003	100	2.966	0.8	28.89	0.0103	10/16.08
	U-O_2	3	2.384 ± 0.019	0.009 ± 0.003	100					
	U-Fe_1	0.5	3.291 ± 0.032	0.006 ± 0.004[†]	97.6					
	U-Fe_2	0.5	3.474 ± 0.039	0.006 ± 0.004[†]	97.4					
	U-U	1	3.710 ± 0.028	0.009 ± 0.003	99.2					
**1 week[c,h]**	U-O_1	3	2.257 ± 0.023	0.0097 ± 0.003	100	4.785	0.9	36.34	0.018	7/14.26
	U-O_2	3	2.403 ± 0.023	0.0075 ± 0.003	100					
	U-U	1	3.815 ± 0.027	0.0058 ± 0.003	99.9					
**3 months[d,e]**	U-O_1	4	2.311 ± 0.010	0.006 ± 0.001	100	6.775	0.9	53.93	0.019	9/18.83
	U-O_2	2	2.463 ± 0.017	0.004 ± 0.002	100					
	U-O_3	8	4.416 ± 0.023	0.009 ± 0.004	100					
	U-U	5	3.861 ± 0.010	0.007 ± 0.001	100					
**6 months[d,f]**	U-O_1	5	2.323 ± 0.009	0.007 ± 0.001	100	6.613	0.9	22.42	0.018	9/19.82
	U-O_2	2	2.475 ± 0.019	0.005 ± 0.002	100					
	U-O_3	14	4.436 ± 0.014	0.009 ± 0.002	100					

U-U	8	3.864 ± 0.008	0.008 ± 0.001	100

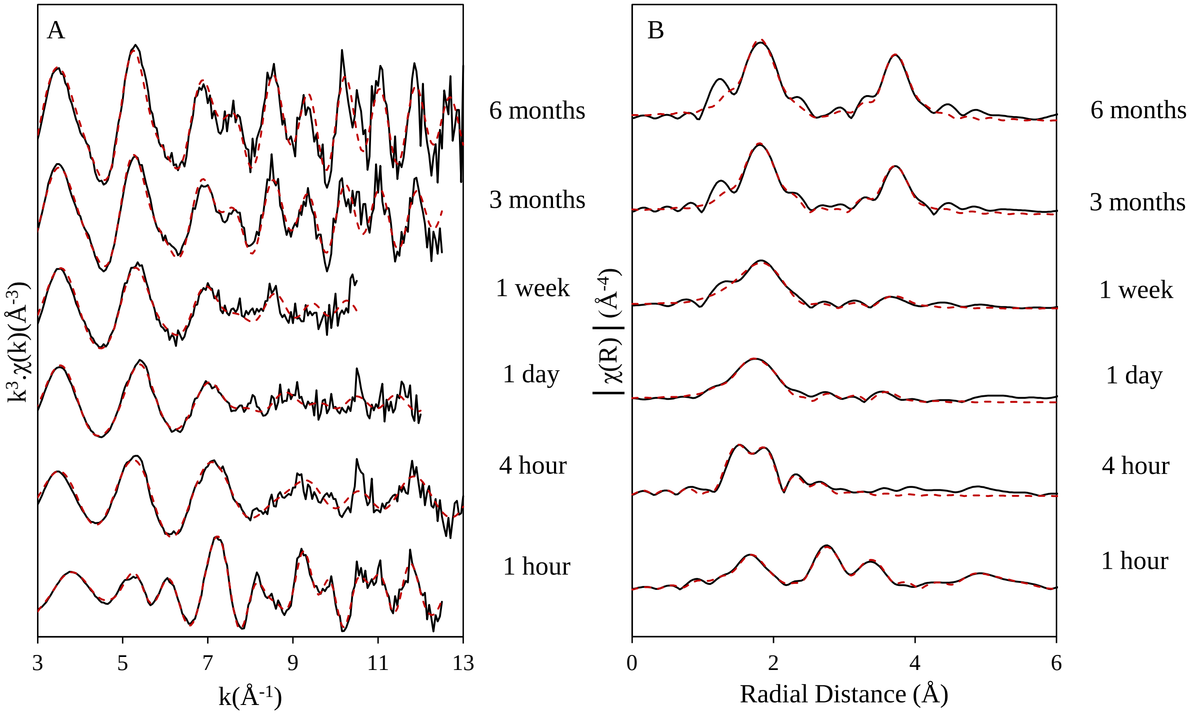

Figure S18. U L_{III}-edge EXAFS (A) and Fourier Transform (B) for the sulfidation U(VI)-goethite.

3.2 Re-oxidation of sulfidised U(VI)-goethite

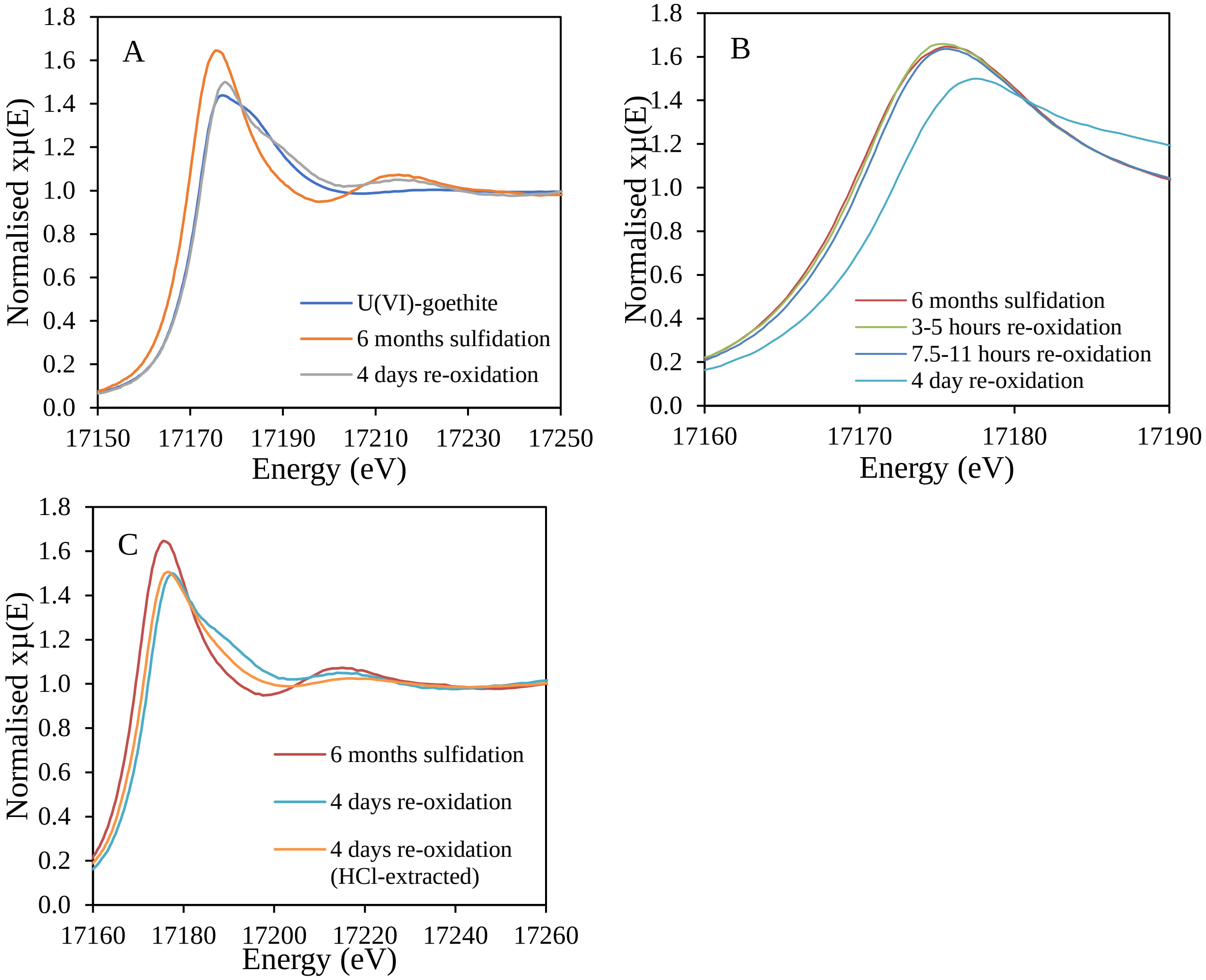

Figure S19. U L_{III}-edge XANES of sulfidised (6 months) U(VI)-goethite and re-oxidised (4 days) U(VI)-goethite, compared to (A) U(VI)-goethite standard; (B) timepoints for re-oxidised U(VI)-goethite (3-5 hours, 7.5-11 hours); (C) HCl-extracted re-oxidised (4 days) U(VI)-goethite.

Table S3. Details of EXAFS best fit parameters from re-oxidised sulfidised U(VI)-goethite. CN denotes coordination number; R denotes the atomic distance; σ^2 denotes the Debye-Waller factor; α denotes the statistical significance of each shell from the F-test, determined from whether the fit was significantly worsened on removal of an individual Fe shell; E_0 denotes the shift in energy from the calculated Fermi level; S_0^2 denotes the amplitude factor; χ_v^2 denotes the reduced χ squared value; R-factor describes the "goodness of fit"; V is the number of variables; NIDP is the number of independent points. All CN and $S0^2$ values are fixed. [†]Indicates the two parameters were linked in a given fit. [a]R range = 1.15 – 4; [b]R range 1.15-3.869; [c]K range = 3-10; [d]K range = 3-13; [e]K range = 3-12.

Sample	Path	CN	R (Å)	σ^2 (Å^2)	α (%)	E_0 (eV)	S_0^2	χ_v^2	R-factor	V/NIDP
**3-5 hours**[a,c]	U-O_1	2	2.186 ± 0.019	0.0025 ± 0.0021	100	2.888	0.9	14.86	0.0208	7/12.49
	U-O_2	4	2.369 ± 0.015	0.004 ± 0.002	100					
	U-U_1	3	3.820 ± 0.018	0.005 ± 0.002	100					
**7.5-11 hours**[a,c]	U-O_1	2	2.209 ± 0.034	0.006 ± 0.005	99.5	4.648	0.9	74.09	0.0203	7/12.49
	U-O_2	4	2.371 ± 0.020	0.006 ± 0.003	100					
	U-U_1	3	3.852 ± 0.021	0.005 ± 0.002	100					
**4 days**[a,d]	U-O_1	2	1.807 ± 0.012	0.004 ± 0.001	100	9.960	0.9	42.77	0.019	9/17.88
	U-O_2	3	2.303 ± 0.031	0.008 ± 0.003	100					
	U-O_3	2	2.454 ± 0.038	0.005 ± 0.004	99.1					
	U-Fe_1	0.5	3.436 ± 0.049	0.006 ± 0.005	89.2					

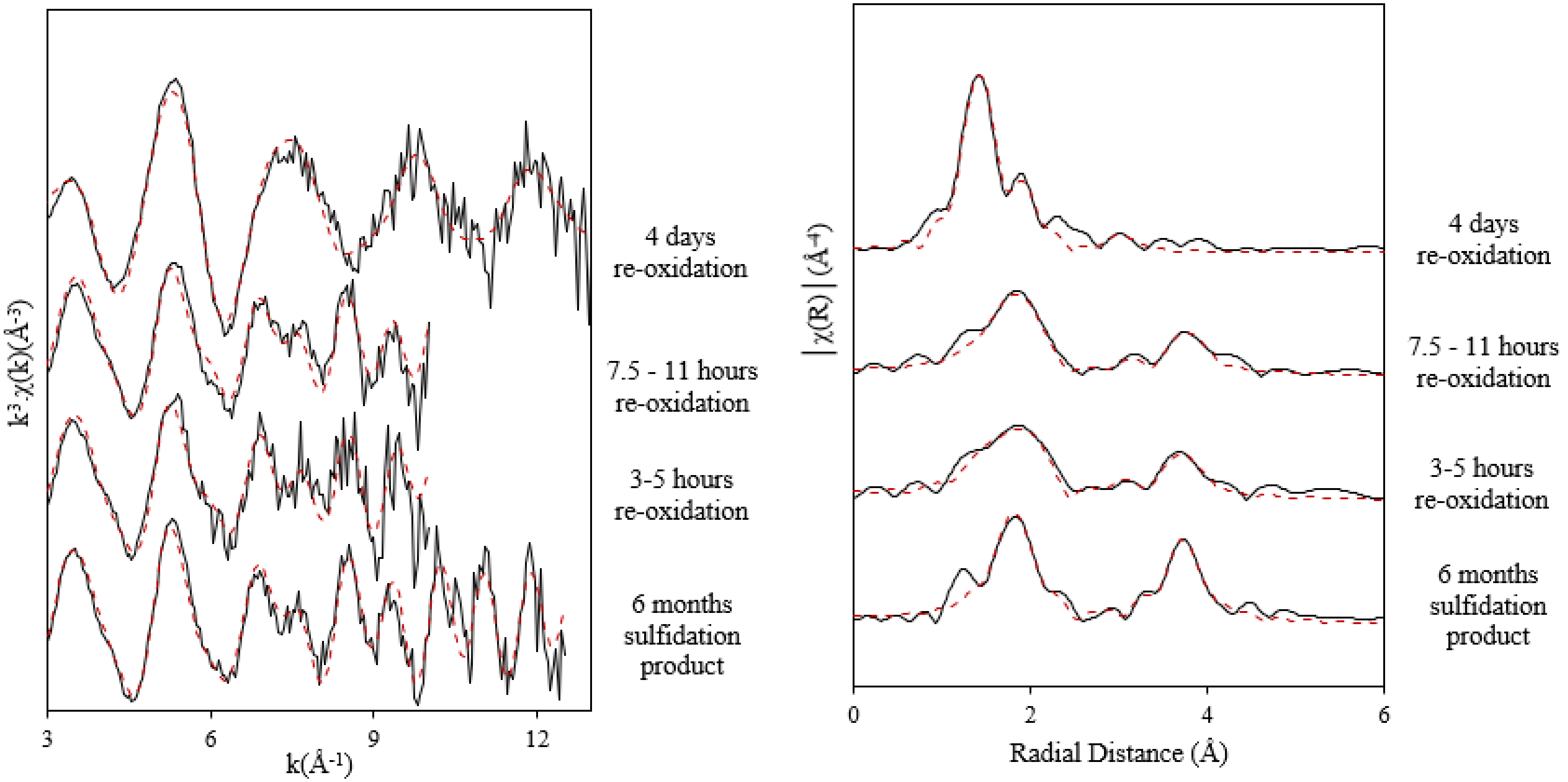

Figure S20. U L_{III}-edge EXAFS (A) and Fourier Transform (B) for the re-oxidised sulfidised U(VI)-goethite.

REFERENCES

(1) Ravel, B.; Newville, M. ATHENA, ARTEMIS, HEPHAESTUS: Data Analysis for X-Ray Absorption Spectroscopy Using IFEFFIT. *J Synchrotron Radiat* **2005**, *12* (4), 537–541.

(2) Scheinost, A. C.; Claussner, J.; Exner, J.; Feig, M.; Findeisen, S.; Hennig, C.; Kvashnina, K. O.; Naudet, D.; Prieur, D.; Rossberg, A.; Schmidt, M.; Qiu, C.; Colomp, P.; Cohen, C.; Dettona, E.; Dyadkin, V.; Stumpf, T. ROBL-II at ESRF: A Synchrotron Toolbox for Actinide Research. *J Synchrotron Radiat* **2021**, *28*, 333–349.

(3) Kvashnina, K. O.; Scheinost, A. C.; Beamline, R. A Johann-Type X-Ray Emission Spectrometer at the Rossendorf Beamline. *J Synchrotron Radiat* **2016**, *23*, 836–841.

(4) Kvashnina, K. O.; Butorin, S. M.; Martin, P.; Glatzel, P. Chemical State of Complex Uranium Oxides. *Phys Rev Lett* **2013**, *111* (25), 253002.

(5) Kvashnina, K. O.; Butorin, S. M. High-Energy Resolution X-Ray Spectroscopy at Actinide M4,5 and Ligand K Edges: What We Know, What We Want to Know, and What We Can Know. *Chemical Communications* **2022**, *58* (3), 327–342.

(6) Roßberg, A.; Reich, T.; Bernhard, G. Complexation of Uranium(VI) with Protocatechuic Acid-Application of Iterative Transformation Factor Analysis to EXAFS Spectroscopy. *Anal Bioanal Chem* **2003**, *376* (5), 631–638.

(7) Vitova, T.; Pidchenko, I.; Schild, D.; Prüßmann, T.; Montoya, V.; Fellhauer, D.; Gaona, X.; Bohnert, E.; Rothe, J.; Baker, R. J.; Geckeis, H. Competitive Reaction of Neptunium(V) and Uranium(VI) in Potassium-Sodium Carbonate-Rich Aqueous Media: Speciation Study with a Focus on High-Resolution X-Ray Spectroscopy. *Inorg Chem* **2020**, *59* (1), 8–22.

(8) Vitova, T.; Pidchenko, I.; Fellhauer, D.; Bagus, P. S.; Joly, Y.; Pruessmann, T.; Bahl, S.; Gonzalez-Robles, E.; Rothe, J.; Altmaier, M.; Denecke, M. A.; Geckeis, H. The Role of the 5f Valence Orbitals of Early Actinides in Chemical Bonding. *Nat Commun* **2017**, *8* (1), 1–9.
